# Role of Flagellin-Homologous Proteins in Biofilm Formation by Pathogenic *Vibrio* Species

**DOI:** 10.1128/mBio.01793-19

**Published:** 2019-08-13

**Authors:** You-Chul Jung, Mi-Ae Lee, Kyu-Ho Lee

**Affiliations:** a Department of Life Science, Sogang University, Seoul, South Korea; University of California, Santa Cruz; University of Washington

**Keywords:** biofilm matrix, flagellin-homologous proteins, *Vibrio*, exopolysaccharides

## Abstract

The pathogenic bacterium Vibrio vulnificus exhibits the ability to form biofilm, for which initiation is dependent upon swimming motility by virtue of a polar flagellum. The filament of its flagellum is composed of multiple flagellin subunits, FlaA, -B, -C, and -D. In V. vulnificus genomes, however, open reading frames (ORFs) annotated by FlaE and -F are also present. Although neither FlaE nor FlaF is involved in filament formation and cellular motility, they are well expressed and secreted to the extracellular milieu through the secretion apparatus for flagellar assembly. In the extrapolymeric matrix of V. vulnificus biofilm, significant levels of FlaEF were detected. Mutants defective in both *flaE* and *flaF* formed significantly decreased biofilms compared to the wild-type biofilm. Thus, the potential role of FlaEF during the biofilm-forming process was investigated by exogenous addition of recombinant FlaEF (rFlaEF) to the biofilm assays. The added rFlaE and rFlaF were predominantly incorporated into the biofilm matrix formed by the wild type. However, biofilms formed by a mutant defective in exopolysaccharide (EPS) biosynthesis were not affected by added FlaEF. These results raised a possibility that FlaEF specifically interact with EPS within the biofilm matrix. *In vitro* pulldown assays using His-tagged rFlaEF or rFlaC revealed the specific binding of EPS to rFlaEF but not to rFlaC. Taken together, our results demonstrate that V. vulnificus FlaEF, flagellin-homologous proteins (FHPs), are crucial for biofilm formation by directly interacting with the essential determinant for biofilm maturation, EPS. Further analyses performed with other pathogenic *Vibrio* species demonstrated both the presence of FHPs and their important role in biofilm formation.

## Abstract (precis)

Flagellar filaments of the pathogenic *Vibrio* species, including V. vulnificus, V. parahaemolyticus, and V. cholerae, are composed of multiple flagellin subunits. In their genomes, however, there are higher numbers of the ORFs encoding flagellin-like proteins than the numbers of flagellin subunits required for filament assembly. Since these flagellin-homologous proteins (FHPs) are well expressed and excreted to environments via a flagellin transport channel, their extracellular role in the pathogenic *Vibrio* has been enigmatic. Their biological significance, which is not related with flagellar functions, has been revealed to be in maturation of biofilm structures. Among various components of the extracellular polymeric matrix produced in the V. vulnificus biofilms, the exopolysaccharides (EPS) are dominant constituents and crucial in maturation of biofilms. The enhancing role of the V. vulnificus FHPs in biofilm formation requires the presence of EPS, as indicated by highly specific interactions among two FHPs and three EPS.

## INTRODUCTION

Mature structures of bacterial biofilms are constructed and maintained by growing the cells initially adhered on the surfaces within the biofilm matrix and then dispersing some cells from biofilm structures. Thus, the extracellular polymeric matrix (EPM) of biofilms, produced by bacteria themselves, is dynamic in its physicochemical characteristics to continually and differentially interact with the ambient bacterial cells (1). The EPM is composed of various macromolecules, including polysaccharides, nucleic acids, and proteins (2). There have been numerous reports showing the specific interactions among the components of biofilm EPM and bacterial cells to ensure the appropriate integrity of biofilms under the given conditions. For example, the capsular polysaccharide (CPS) produced by Vibrio vulnificus is required for the dispersal stage of biofilm formation by decreasing the hydrophobicity in the mature EPM environments (3). A major polysaccharide of Pseudomonas aeruginosa, Pel, is a cationic exopolysaccharide (EPS) that interacts with extracellular DNA in the biofilm matrix to provide structural integrity to biofilms (4). Matrix proteins of Vibrio cholerae biofilms play roles in intercellular interactions by facilitating bacterial aggregation and encasing cell clusters (5).

For timely expression of EPM components during the specific stages of biofilm formation or under specific conditions, bacterial cells utilize diverse signal recognition systems and subsequent regulatory mechanisms, for example, quorum sensing (6–8). Another signal molecule, cyclic di-GMP, also elicits pleiotropic effects on biofilm formation by diverse bacterial species via regulating flagellar formation or motion (7). Motility mediated by flagellum has been shown to be important in initiation of biofilm formation by increasing the probability of bacterial encounters with the surfaces (7). In addition, an Escherichia coli flagellum was shown to provide physical frames in biofilm EPM (9).

The flagellum, a helical propeller rotating by a reversible rotary motor, confers swimming motility to bacteria. It is composed of three core structures, the basal body, the hook, and the long and thin filament. The basal body, including a rotor and a stator, is embedded in the bacterial membrane (10). The hook refers to a joint connecting the basal body and the outward bound filament. A hook is composed of more than a hundred subunits of FlgE (10). The hook-associated proteins (HAPs), i.e., FlgK (HAP1) and FlgL (HAP3), are structural adaptors between the flexible hook and the rigid filament (10). A filament composed of up to 20,000 subunits of flagellins is capped at the end of its structure with the scaffolding protein FliD (HAP2) (10). The bacterial flagella show extensive variation among species in terms of their numbers, extracellular localities, and usages (10).

**FIG 1:**
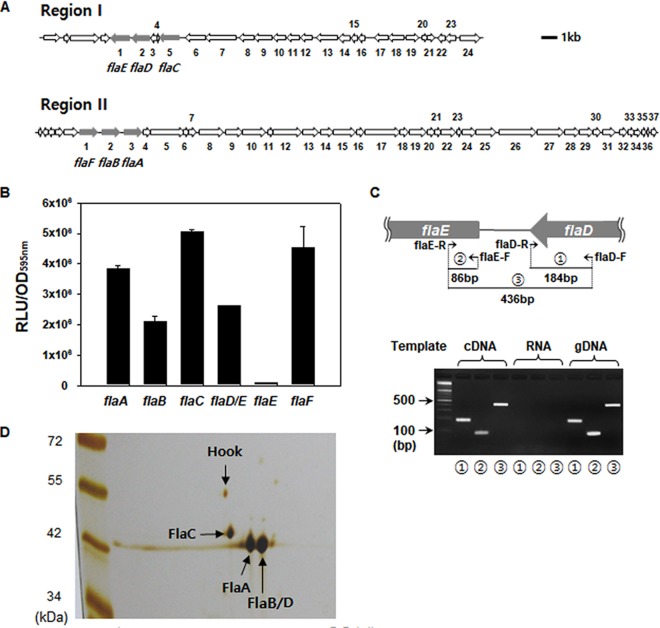
Transcription of flagellin-like ORFs in V. vulnificus MO6-24/O and composition of the flagellar filament. (A) V. vulnificus MO6-24/O genomes include six flagellin-like ORFs, VVM06_00807 (*flaF*), VVM06_00808 (*flaB*), VVM06_00809 (*flaA*), VVM06_02251 (*flaE*), VVM06_02252 (*flaD*), and VVM06_02255 (*flaC*). *flaCDE* and *flaFBA* are located at region I and region II, respectively. The information for the ORFs in region I (numbered 1 through 24) and those in region II (numbered 1 through 37) is provided in the Table S1. (B) Wild-type strains carrying a transcriptional reporter (*luxAB*) plasmid inserted by the upstream region of each flagellin gene were grown in AB-fumarate medium supplemented with 3 μg/ml of tetracycline. At exponential phase, aliquots were sampled to determine the cell masses (OD_595_) and bioluminescence (relative light units [RLU]). Luciferase activities were expressed as normalized values by dividing the RLU by the OD_595_ of each sample. (C) RT-PCR. cDNA which had been produced through the reverse transcriptase reaction of total RNA extracted from wild-type V. vulnificus was subjected to PCR using three sets of primers. Expected sizes of PCR products (products 1, 2, and 3) are 184, 86, and 436 bp, respectively. Genomic DNA (gDNA) or RNA extracts were used as templates to serve as the positive or negative controls for PCR, respectively. (D) FlaA, FlaB/D, and FlaC. A flagellar fraction was harvested from the wild-type V. vulnificus grown in AB-fumarate medium for 16 h, and 10 μg of flagellar preparation was subjected to 2-D gel analysis. The protein spots, which were separated through isoelectric focusing and SDS-PAGE and then detected by silver staining, were identified via MALDI-TOF analysis and are designated by arrows with the names of flagellins (FlaA, -B/D, and -C) or hook (FlgE).

Among the flagellar components listed above, a great diversity has been reported in the filament’s composition. The genomes of motile bacteria having flagella show variations in the arrangement and numbers of the open reading frame (ORFs) encoding flagellin(s). A single flagellin gene is present in the genomes of E. coli, Pseudomonas aeruginosa, and Bacillus subtilis (11–13). In contrast, *Salmonella* species have two copies of flagellin genes (14), and *Vibrio* species have ∼5 or 6 flagellin genes. Six flagellin ORFs are found in Vibrio fischeri chromosomes as two clusters of *flaABCDE* and *flaF* (15), and five flagellin ORFs are in V. cholerae chromosomes as two clusters of *flaAC* and *flaEDB* (16). In Vibrio parahaemolyticus, six flagellin ORFs are present as two clusters of *flaFBA* and *flaCDE* (17). Another foodborne pathogen, V. vulnificus, shows the same number and arrangement of ORFs annotated by flagellin genes as V. parahaemolyticus. Among the flagella of V. fischeri, V. cholerae, and V. vulnificus, only ∼4 or 5 kinds of flagellins have been identified to constitute their filaments (15, 18, 19). From our preliminary investigation of the filament constituents of V. vulnificus strain MO6-24/O, four flagellins, FlaABCD, were present in its flagellar preparation (see Fig. 1). However, the other two flagellin-homologous proteins (FHPs), FlaEF, which are absent from flagellar structure, were found to be highly accumulated in the culture supernatants. These preliminary findings led us to speculate the unique role(s) of these FHPs in the extracellular environment. Therefore, in this study, we investigated why FHPs are not involved in filament assembly, what the secreted FHPs do, and how FHPs specifically achieve their function in extracellular environments. Furthermore, our understanding of FHPs was expanded by examining the other pathogenic *Vibrio* species.

10.1128/mBio.01793-19.4 TABLE S1 Gene numbers and annotation of ORFs in regions I and II of flagellar assembly in V. vulnificus MO6-24/O. Download Table S1, PDF file, 0.2 MB. Copyright © 2019 Jung et al. 2019 Jung et al. This content is distributed under the terms of the Creative Commons Attribution 4.0 International license.

## RESULTS

### Transcription of the putative flagellin ORFs in V. vulnificus and identification of its filament constituents.

The genome of V. vulnificus MO6-24/O contains at least six ORFs tentatively constituting flagellin genes, annotated by *flaA* to *flaF*. These putative flagellin genes are located in region I and region II (see Table S1 in the supplemental material) as gene arrangements *flaC-*(hypothetical ORF)_2_-*flaD-flaE* and *flaF-flaB-flaA*, respectively (Fig. 1A). To investigate the transcription level of these genes, the upstream regions of each flagellin gene, *flaA*, *flaB*, *flaC*, *flaD*, *flaE*, and *flaF*, were used for constructing *luxAB*-based transcriptional reporter plasmids. The wild-type strain carrying each flagellin gene fusion was grown in AB-fumarate medium (see Materials and Methods) supplemented with 3 μg/ml of tetracycline, and their expressions were monitored. The resultant transcription reporters showed that all the putative flagellin gene fusions, except for the *flaE-luxAB* fusion, were well transcribed (Fig. 1B). As reported for V. parahaemolyticus (17), the *flaE* ORF appeared to be transcribed from the promoter located in the upstream region of the *flaD* ORF in V. vulnificus. *In silico* analysis of the intergenic space between *flaD* and *flaE* showed no apparent promoter, but the RpoF-dependent promoter was discernible in the upstream region of *flaD*. In addition, the presence of a transcript encompassing both *flaD* and *flaE* was confirmed by reverse transcriptase PCR (RT-PCR) (Fig. 1C). Amplification of a DNA fragment containing the intergenic space between *flaD* and *flaE* (designated by a circled “3” in Fig. 1C) was successful in the PCR using cDNA prepared from the total RNA of wild-type V. vulnificus. This indicated that *flaE* was cotranscribed with its upstream *flaD* gene and six ORFs encoding the putative flagellins were well transcribed in V. vulnificus.

To examine the flagellin subunits of V. vulnificus, purified filaments were subjected to two-dimensional (2-D) gel electrophoresis (Fig. 1D). It showed three major protein spots, which were subsequently identified as FlaA, FlaB/D, and FlaC via matrix-assisted laser desorption ionization–time of flight (MALDI-TOF) analysis. The amino acid sequences of FlaB and FlaD are identical in V. vulnificus strains (Fig. S1A and B). A minor spot, for which the molecular weight (MW) and pI were approximately 46.8 kDa and 4.54, respectively, was identified to be FlgE, a subunit of the hook structure. However, neither FlaE nor FlaF was detected in this 2-D gel analysis of the V. vulnificus flagellum. This observation suggested that the filament of the V. vulnificus flagellum was composed of four flagellins, FlaA, FlaB/D, and FlaC, but did not include FlaE and FlaF.

10.1128/mBio.01793-19.1 FIG S1 Flagellins (FlaA, -B, -C, and -D) and flagellin-homologous proteins (FlaE and -F) of V. vulnificus. (A) Sizes of FlaA to -F. ORFs of *flaA* to *-F* are composed of 1,128 to 1,158 bp. Based upon the nucleotide sequences, the expected molecular weights and pIs of each protein were calculated. (B) Pairwise comparison of the amino acid sequences of FlaA to -F. Homologies among FlaA to -F are presented with the percentages of identical amino acids in pairwise alignments of six proteins by using the NCBI Basic Local Alignment Search Tool. (C) Purified recombinant proteins of FlaA to -F (2 μg) were run on SDS-PAGE and stained with Coomassie blue. The calculated sizes (in kilodaltons) of each recombinant protein are provided. (D) Western blot analysis of FlaA to -F. Purified recombinant proteins of FlaA to -F (0.1 μg) were run on SDS-PAGE and subjected to Western blotting using the anti-Fla polyclonal antibodies. Please note that the antibodies used in this study equally reacted with all six recombinant proteins. Download FIG S1, TIF file, 1.4 MB. Copyright © 2019 Jung et al. 2019 Jung et al. This content is distributed under the terms of the Creative Commons Attribution 4.0 International license.

### Production and secretion of FlaE and FlaF.

**FIG 2:**
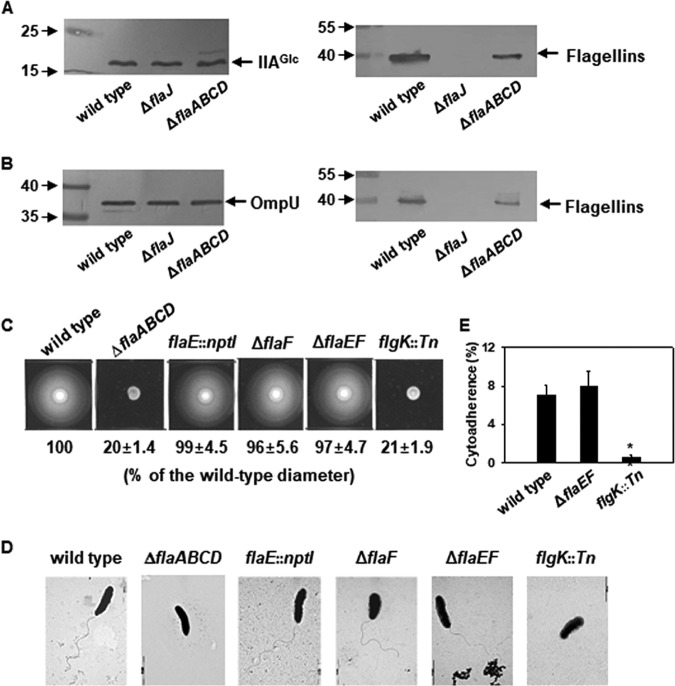
Production of FlaEF and their roles in motility and flagellation of V. vulnificus. (A and B) FlaE and FlaF. Cell lysates and the culture supernatants were prepared from the wild-type, Δ*flaABCD*, and Δ*flaJ* strains of V. vulnificus. Sixty micrograms of crude cell extracts (A) and 2 μg of the concentrated proteins in culture supernatants (B) were subjected to SDS-PAGE and then Western blot analysis using polyclonal antibodies reacting with all the flagellin-like proteins from FlaA to FlaF. As loading controls for cell lysate and culture supernatant, the antibodies specific to IIA^Glc^ and OmpU were utilized, respectively. The Δ*flaJ* strain was included as a negative control in this assay. Each immunoreactive band detected by specific antibodies is indicated with an arrow. Numbers on the left sides of the blots are molecular weight markers, in kilodaltons. (C) Motility on a soft agar plate. Fresh cultures of Δ*flaABCD*, *flaE*::*nptI*, Δ*flaF*, and Δ*flaEF* strains were spotted onto a soft agar (0.3%) plate and incubated at 30°C for 6 h. To compare the degrees of bacterial motility, wild-type and *flgK*::*Tn* strains were included in this assay as positive and negative controls, respectively. The motility of each mutant is shown as the percentage of the colony diameters made by the wild type. (D) Electron microscopic observation of the polar flagellum. Fresh cultures of each V. vulnificus strains were treated as described in Materials and Methods to observe the flagella using TEM, harvested, and resuspended in 300 μl of PBS. (E) Adherence to a host cell. INT-407 cells were exposed to wild-type, Δ*flaEF*, or *flgK*::*Tn* strains of V. vulnificus at an MOI of 10. After 30 min of incubation, attached bacterial cells were recovered and then plated on an LBS agar plate. Measured numbers of adhering bacterial cells are presented as the percentage of the bacterial cells added to the cytoadherence assay.

Although transcriptions of both *flaE* and *flaF* genes were observed, it was not clear whether their translation occurred in V. vulnificus. To examine the production of FlaE and FlaF, their contents in the cell lysates (Fig. 2A) and culture supernatants (Fig. 2B) were observed by using polyclonal antibodies reacting with FlaA to -F. Since the homologies among four flagellins, FlaE, and FlaF are high (∼53.2 to 75.4% identities [Fig. S1B]), the antibodies raised against any flagellin, FlaE, or FlaF showed cross-reaction with all six proteins (Fig. S1C and D). Thus, to differentiate the FlaA/B/C/D and FlaE/F, two mutant strains which were unable to produce FlaA/B/C/D (Δ*flaABCD* mutant) or maintain FlaA/B/C/D/E/F (Δ*flaJ* mutant [20]) were compared with the wild type. FlaJ (or FliS) is known as an essential chaperone for flagellins in the cytoplasm, and thus, no flagellin subunit was expected to be present in either intracellular or extracellular samples if the *flaJ* gene was knocked out. Crude cell lysates and culture supernatants were prepared from three strains, and equal amounts of proteins in each preparation, which were verified by Western blot analyses using the antibodies specific to a cytoplasmic protein (IIA^Glc^ [21]) (left blot in Fig. 2A) or a typical exoprotein (OmpU [22]) (left blot in Fig. 2B), were subjected to Western blot analysis using antibodies reacting with FlaA to -F. As expected, no band was observed in the Δ*flaJ* mutant. In contrast, the presence of a band corresponding to the approximate size (ranging from 39.9 to 41.1 kDa [Fig. S1A]) of flagellins or FlaE/F showed that they were produced in the cytoplasm of the Δ*flaABCD* mutant (right blot in Fig. 2A) and secreted to culture media by the Δ*flaABCD* mutant (right blot in Fig. 2B). It is believed that this band represents FlaE and/or FlaF because the intensities of such immunoreactive bands from the Δ*flaABCD* mutant were less than those of wild type producing all the six proteins. These results indicated that FlaE and/or FlaF was translated and then secreted.

To identify the function of FlaE and FlaF in V. vulnificus, the typical phenotypes derived from the functional polar flagellum were investigated using the mutant V. vulnificus defective in production of FlaE and/or FlaF, e.g., the *flaE*::*nptI* and Δ*flaF* mutants and the Δ*flaEF* double mutant. The swimming motility of these mutants was compared to those of the wild type and two nonflagellated mutants, the *flgK*::*Tn* and Δ*flaABCD* mutants (Fig. 2C and D). All the mutant strains except for the *flgK*::*Tn* and Δ*flaABCD* mutants showed the same degree of swimming motility as the wild type (Fig. 2C). Observation of the wild-type and mutant cells under a transmission electron microscope (TEM) revealed that all the strains, except for the *flgK*::*Tn* and Δ*flaABCD* mutants, had the polar flagellar structure (Fig. 2D). This result is consistent with the results of 2-D gel electrophoresis of the filament proteins, which showed that no FlaE/F was required for construction of a filament structure (Fig. 1D). In addition to bacterial motility, the flagella of pathogenic bacteria have been found to be involved in adherence to host cells (23). Thus, the possible role of FlaE and FlaF in bacterial cytoadherence was examined by challenging the wild-type, Δ*flaEF*, and *flgK*::*Tn* strains against a human epithelial cell line, INT-407, for 30 min at a multiplicity of infection (MOI) of 10 (Fig. 2E). As expected, the nonmotile *flgK*::*Tn* mutant showed a significant decrease in cytoadherence to INT-407 (*P* < 0.005, Student’s *t* test). In contrast, for the Δ*flaEF* mutant 7.9% of the inoculum cell concentrations were adhered, which was the same level of adherence to the host cells exhibited by the wild type. These findings on motility, flagellation, and cytoadherence demonstrated that FlaE and FlaF were not involved in structure or function of the polar flagellum of V. vulnificus.

### Identification of a secretion pathway for the FHPs, FlaE and FlaF.

**FIG 3:**
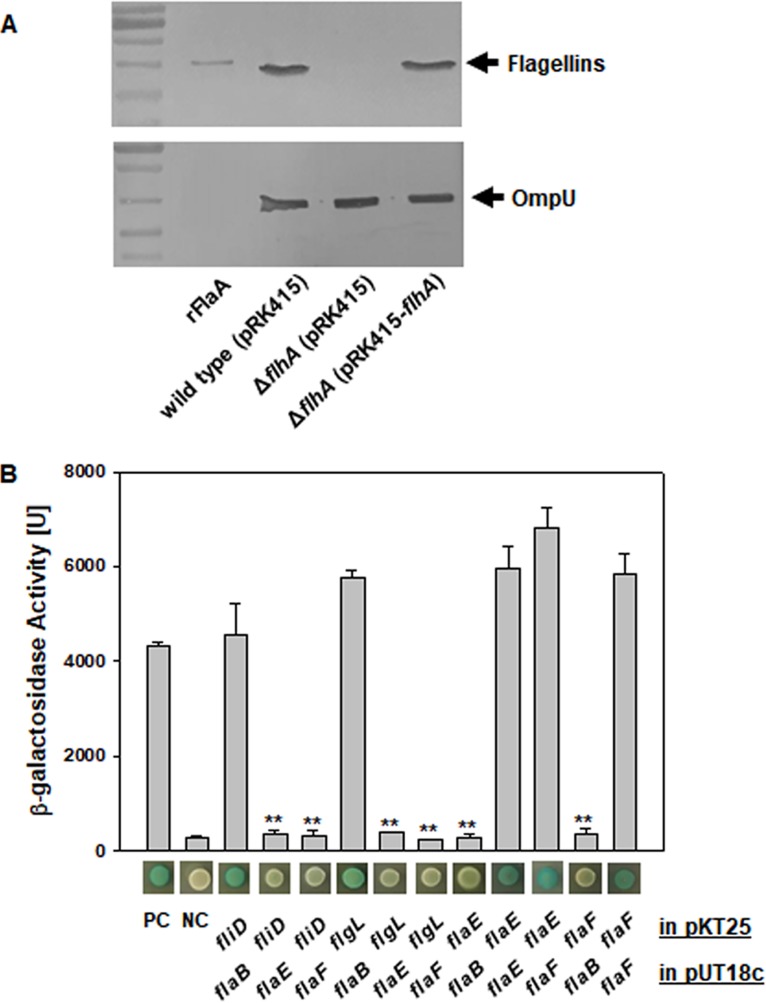
Extracellular secretion of FlaE and FlaF and their interaction with the flagellar components. (A) Role of FlhA in secretion of FlaE and -F. Protein pools in the culture supernatants of the wild type, Δ*flhA* mutant, and Δ*flhA* mutant complemented with the intact *flhA* gene were used for Western blot analysis using polyclonal antibodies reacting with all the flagellin-like proteins from FlaA to FlaF. An immunoreactive band of the flagellins (FlaABCD) and flagellin-homologous proteins (FHPs; FlaEF), as referenced by a band derived from recombinant FlaA (rFlaA), is indicated with an arrow. As a loading control, the same samples were also exposed to antibodies specific to OmpU. (B) Interaction of FlaE and FlaF with flagellar components. To examine the degree of specific interaction of FHPs with each other and other flagellar components, a two-hybrid system showing β-galactosidase activity was utilized (bacterial two-hybrid system kit). The genes cloned into pUT18C or pKT25 (*flaE*, *flaF*, *flaB*, *fliD*, and *flgL*) are designated below the bar graph. The β-galactosidase activities are exhibited by blue color developed on the colonies grown on X-Gal agar plates and quantified by enzyme assay catalyzing the conversion of ONPG. The positive (PC) and negative (NC) controls for β-galactosidase assays are E. coli BTH101 cells containing pUT18c-*zip*/pKT25-*zip* and pUT18c/pKT25, respectively.

As shown in Fig. 2B, FlaE and/or FlaF was found in the culture supernatants of the wild type and Δ*flaABCD* mutant. Since flagellins have been reported to be secreted via interactions with an export gate protein, FlhA (24), a Δ*flhA* mutant strain of V. vulnificus was constructed. Western blotting showed that this mutant did not show any flagellin-sized immunoreactive band, which was regained in the mutant strain complemented with the intact *flhA* gene (Fig. 3A). This result implied that FlaE and/or FlaF was also secreted through FlhA as the flagellins are. It is proposed that FlaE/F are not involved in the subsequent series of processes of filament construction, although they share a secretion channel with the flagellin subunits, FlaA/B/C/D. Thus, it might be hypothesized that FlaE and FlaF did not interact with other components of filament during the period of secretion to the extracellular milieu. To examine this hypothesis, a β-galactosidase-based bacterial two-hybrid system (BACTH) was utilized. Various combinations of two plasmids containing the genes encoding one of the filament components (i.e., *flaB*, *flgL*, and *fliD*) and either of the genes *flaE* and *flaF* were prepared and transferred to an E. coli host (Fig. 3B). The β-galactosidase activities of E. coli cells carrying each combination of two plasmids were measured and compared with those of the positive-control (PC) and negative-control (NC) cells. As expected from the previous findings regarding the interactions of HAPs in other bacterial species (25, 26), a flagellin subunit, FlaB, showed strong interactions with FlgL (HAP3) and FliD (HAP2). In contrast, neither FlaE nor FlaF interacted with FlaB, FlgL, or FliD. Instead, FlaE and FlaF showed strong interaction among themselves or each other at the degree of protein-protein interaction shown by the PC. This result could explain the reason why FlaE and FlaF were not retained in filament structure but were secreted to the extracellular milieu through flagellin secretion channel. To elucidate any unidentified function(s) of the secreted flagellin-homologous proteins (FHPs), their role in forming the mature biofilms was tested by performing biofilm assays in the absence of FlaE and/or FlaF.

### Role of FHPs in biofilm formation.

**FIG 4:**
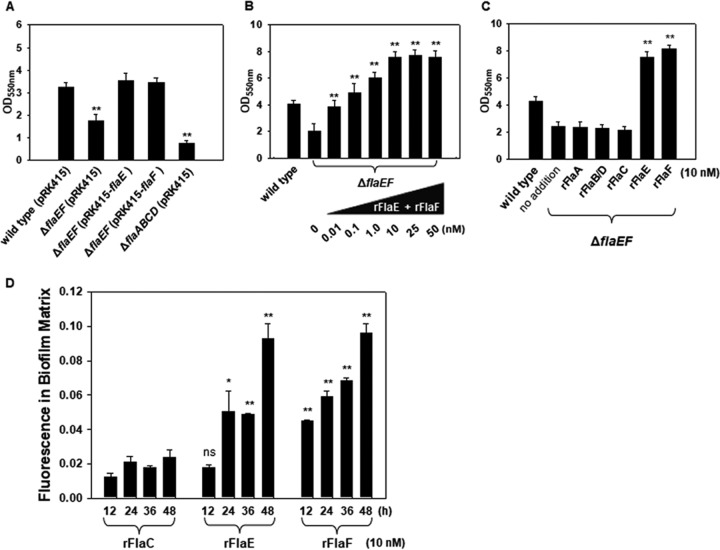
Biofilm-forming ability of the Δ*flaEF* mutant and the effect of FlaEF addition on biofilm formation. (A) Effect of *flaEF* deletions on biofilm formation. A standard biofilm assay using borosilicate tubes was performed with the wild type carrying pRK415, the Δ*flaEF* mutant carrying pRK415, the Δ*flaEF* mutant carrying pRK415-*flaE*, the Δ*flaEF* mutant carrying pRK41-*flaF*, and the Δ*flaABCD* mutant carrying pRK415. After incubation at 30°C for 48 h, biofilm growth was estimated by staining with crystal violet and subsequent spectrophotometry at 550 nm (OD_550_). The *P* values for comparison with the wild type are indicated (*, 0.005 ≤ *P < *0.05; **, *P < *0.005). (B) Effect of rFlaEF addition on the biofilm formation. Biofilm growth of the Δ*flaEF* mutant was monitored in the absence and presence of the recombinant proteins rFlaE and rFlaF at concentrations ranging from 0.01 to 50 nM. Incubation condition and biofilm estimation were as described above. (C) Effect of addition of flagellins on biofilm formation. Biofilm growth of the Δ*flaEF* mutant was monitored in the presence of 10 nM concentrations of rFlaA, rFlaB, and rFlaC. For comparison, the same concentrations of rFlaE and rFlaF were included in the assay. Incubation condition and biofilm estimation were as described above. (D) Incorporation of FlaEF in biofilms. rFlaC, rFlaE, and rFlaF were labeled with Alexa Fluor 555, and then the same amounts of labeled protein (10 nM) were added to the biofilm assay wells inoculated with the Δ*flaEF* mutant. At every 12 h, the fluorescent intensities, the excitation at 555 nm and the emission at 565 nm, were measured from the resuspended biofilms. Fluorescence in biofilm matrix derived from the labeled proteins associated with biofilms is presented as the percentage of the fluorescence from the initially added labeled protein. ns, not significant.

The abilities of the Δ*flaEF* mutant carrying pRK415, the Δ*flaEF* mutant carrying pRK415-*flaE*, and the Δ*flaEF* mutant carrying pRK415-*flaF* to form biofilms were compared with those of the wild type carrying pRK415 and the nonmotile Δ*flaABCD* mutant carrying pRK415 (Fig. 4A). Biofilm formation by another nonmotile mutant of V. vulnificus, in which the *flgE* gene was knocked out, has been previously shown to be minimal (27). Similarly, the Δ*flaABCD* mutant showed about 23.1% of the biofilm formation of the wild type. The degree of biofilm formation by the Δ*flaEF* mutant was estimated to be about 53.7% that of the wild type; however, the mutant exhibited restored biofilm-forming ability, comparable to that of the wild type, when either the *flaE* or *flaF* gene was supplied in *trans*. To verify that the increased biofilm formation by the complemented strains of the Δ*flaEF* mutant was due to production and secretion of FlaE or FlaF, the recombinant FHPs (rFHPs) were purified and exogenously added to the biofilm assay tubes inoculated with the Δ*flaEF* mutant (Fig. 4B). In the presence of various concentrations of rFHPs ranging from 0.01 to 50 nM (equal concentrations of rFlaE and rFlaF), the degrees of biofilm formation by the Δ*flaEF* mutant increased in a concentration-dependent manner up to 10 nM. In the presence of 10 nM (each) rFlaE or rFlaF, the sizes of biofilms formed by the Δ*flaEF* mutant were similar to those formed in the presence of both recombinant proteins (Fig. 4C). To test if the flagellins have the same effect on biofilm formation, the recombinant flagellins (Fig. S1C) were exogenously added to the biofilm assay tubes inoculated with the Δ*flaEF* mutant at a concentration of 10 nM (Fig. 4C). However, there was no difference in biofilm growth in the presence of rFlaA, rFlaB, or rFlaC: the biofilms were almost the same sizes as biofilms formed in the absence of any recombinant protein.

Next, localization of the added rFHPs in biofilm assay tubes were examined using recombinant proteins labeled with Alexa Fluor 555 and monitoring the fluorescence during the incubation period (up to 48 h). At designated time points, biofilms of the Δ*flaEF* mutant were separated from the planktonic biomass, and then the fluorescent dyes associated with the biofilms were quantified (Fig. 4D). Compared to the labeled rFlaC, significant amounts of the labeled rFlaE and rFlaF were localized in biofilm structures. Labeled rFHPs increased in an incubation time-dependent manner, while the labeled rFlaC was not dramatically increased, at least up to 48 h. When the associated fluorescence-labeled rFHPs were normalized by the total fluorescence added at the beginning of incubation, the associated fluorescence at 48 h were 0.024 ± 0.004, 0.093 ± 0.009, and 0.096 ± 0.006 in biofilms formed in the presence of rFlaC, rFlaE, and rFlaF, respectively. These results led us to speculate the potential function of FHPs in maturing biofilms by interaction with EPM of biofilms.

### Association of FHPs in biofilm matrix.

**FIG 5:**
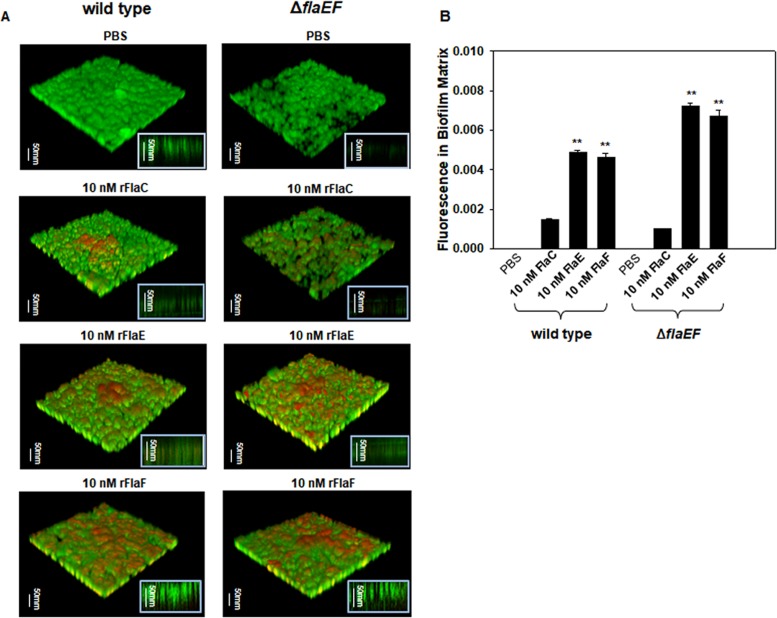
Association of the added rFlaE and rFlaF with biofilms. The wild type carrying pRK415-*gfp* and the Δ*flaEF* mutant carrying pRK415-*gfp* were inoculated to dishes equipped with coverslipped bottoms to form biofilms. Fluorescent dye (Alexa Fluor 555)-labeled rFlaC, rFlaE, and rFlaF (10 nM each) were added at the beginning of incubation. At 48 h, biofilms and their EPM were observed using a confocal microscope. Bacterial cells and labeled proteins in EPM were presented as green and orange colors, respectively (A). The fluorescence in biofilm matrix derived from the Alexa Fluor 555-labeled proteins associated with biofilms was normalized by dividing by the green fluorescence derived from bacterial cells harboring pRK415-*gfp* (B).

To visualize the incorporated FHPs in biofilms, the orange fluorescence-labeled proteins were exogenously added to the biofilm assay vessels inoculated with *gfp*-tagged V. vulnificus wild-type and Δ*flaEF* strains. At 48 h postinoculation, the formed structures of biofilms were observed using a confocal microscope (Fig. 5A). Bacterial biomasses in the biofilms formed by the Δ*flaEF* mutant were estimated by measuring the green fluorescence. There were about 1.7 times more cells in biofilms treated with rFlaE or rFlaF than in those treated with rFlaC. Recombinant proteins associated with biofilms were simultaneously estimated by measuring the fluorescence (Alexa Fluor 555) labeling the recombinant proteins. There was 11 to 12 times more Alexa Fluor 555 in rFlaE- or rFlaF-treated biofilms than in rFlaC-treated biofilms (*P* < 0.005, Student’s *t* test). The normalized levels of fluorescence in Δ*flaEF* biofilms, which were determined as the biofilm-associated Alexa Fluor 555 divided by the green fluorescence of bacterial biomass, were about 0.0010, 0.0072, and 0.0067 in rFlaC, rFlaE, and rFlaF, respectively (Fig. 5B). Thus, as shown in Fig. 4D, approximately 7 times more rFlaE and rFlaF were incorporated in the Δ*flaEF* biofilms than rFlaC. Incorporation of fluorescence-labeled rFlaE and rFlaF was also apparent in the wild-type biofilms, where the amounts of incorporated fluorescence were about 67.5 to 68.7% of the normalized fluorescence in Δ*flaEF* biofilms. These results suggested that FHPs acted as enhancing factors in biofilm formation via interaction with some components of biofilm EPM.

### Interaction of FHPs with major components of biofilm matrix, EPSs.

**FIG 6:**
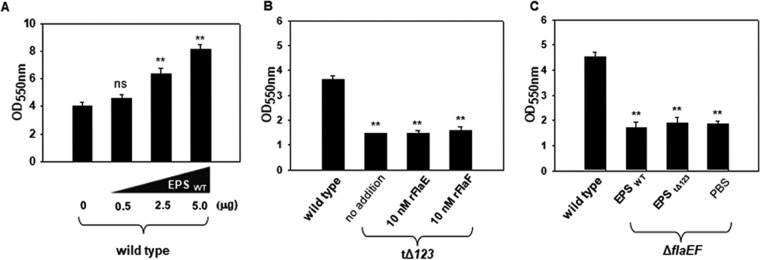
Effects of exogenous addition of rFlaEF and EPS on biofilm formation. (A) Effect of exogenous addition of EPS on biofilm formation. An EPS fraction was prepared from the wild-type cells (EPS_WT_) and various amounts of EPS_WT_, ranging from 0 to 5 μg of glucose equivalents, were added to the biofilm assay using the wild type. After 48 h, biofilm growth was estimated as described for Fig. 4A. (B) Exogenous addition of rFlaEF to an EPS-deficient mutant. In the beginning of biofilm formation by a mutant V. vulnificus defective in the three EPS gene clusters 1, 2, and 3 (tΔ*123* [27]), 10 nM rFlaE or rFlaF was added. Incubation condition and biofilm estimation were as described for Fig. 4A. (C) Exogenous addition of EPS to the Δ*flaEF* mutant. EPS fractions were prepared from the wild type and tΔ*123* strains and then dissolved in PBS. Five micrograms of EPS, which was estimated as glucose-equivalent amounts, was added to the biofilm assay using the Δ*flaEF* mutant.

The most abundant components in the biofilm EPM are extracellular polysaccharides (1). To study the enhancement mechanism of FHPs in biofilm formation, we examined the occurrence of biofilm enhancement in the presence of FHPs exogenously added to mutant strains of V. vulnificus, including a lipopolysaccharide (LPS)-deficient Δ*gmhD* mutant (28) and a capsule (CPS)-deficient strain of the *wbpP*::*Tn* mutant (3). However, the degrees of biofilm formation by the Δ*gmhD* and *wbpP*::*Tn* mutants were increased upon exogenous addition of FHPs (Fig. S2). This indicated that LPS or CPS is not a counterpart for the interaction with FHPs in biofilm matrix. Therefore, further study would focus on the exopolysaccharides (EPSs) of V. vulnificus, which are of at least three kinds, *viz.*, EPS_I_, EPS_II_, and EPS_III_, which are produced by three EPS biosynthesis gene clusters separated in its genome (29). As previously reported (29), the EPSs are critical in biofilm formation by V. vulnificus. Exogenous addition of EPS extracted from wild-type V. vulnificus (EPS_WT_) actually led to increasd biofilm sizes in a dose-dependent manner (Fig. 6A). A mutant deficient in production of EPS_I_, EPS_II_, and EPS_III_, namely, CBtΔ*123* (29), was used to examine the effect of FHPs on biofilm formation (Fig. 6B). The degree of biofilms formed by CBtΔ*123* was not affected by addition of 10 nM FHPs, suggesting that FHPs required the presence of EPS in order to enhance biofilm formation.

10.1128/mBio.01793-19.2 FIG S2 Effect of exogenous addition of rFlaEF on biofilm formation by LPS-deficient and CPS-deficient mutants of V. vulnificus. Various concentrations of rFlaEF, ranging from 0 to 50 nM, were added to the biofilm assays inoculated with an LPS-deficient Δ*gmhD* mutant (A) or a CPS-deficient *wbpP*::*Tn* mutant (B). Incubation conditions and biofilm estimation (OD_550_) were as described for Fig. 4A. Download FIG S2, TIF file, 1.1 MB. Copyright © 2019 Jung et al. 2019 Jung et al. This content is distributed under the terms of the Creative Commons Attribution 4.0 International license.

To verify this preliminary interpretation, a biofilm assay was performed by exogenously adding the EPS fractions extracted from the wild type or CBtΔ*123* to the Δ*flaEF* mutant (Fig. 6C). The degree of biofilm formation by the Δ*flaEF* mutant was not influenced by addition of EPS_WT_ or EPS_tΔ_*_123_* at all: the size was comparable to those of control biofilms. These basal levels of biofilm formation by CBtΔ*123* (optical density at 550 nm [OD_550_] ranging from 1.5 to 1.6 in Fig. 6B) were almost the same as those for the Δ*flaEF* mutant (OD_550_ ranging from 1.7 to 1.9 in Fig. 6C). These results confirmed that the increased biofilm formation via FHPs essentially required the presence of EPS.

### Specific interaction of FlaE and FlaF with EPS_I_/EPS_III_ and EPS_II_, respectively.

**FIG 7:**
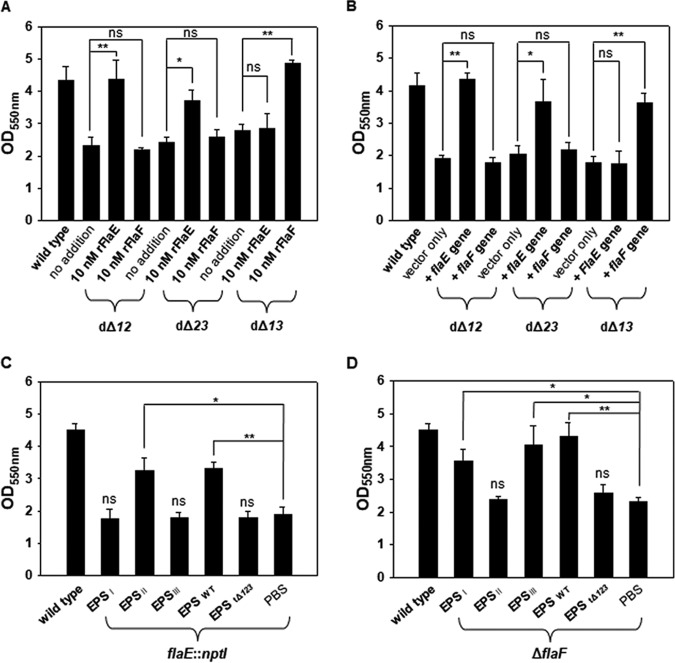
Effect of addition of various EPS on biofilm formation by the *flaE*::*nptI* and Δ*flaF* mutants. (A) Exogenous addition of rFlaE and rFlaF to various EPS mutants. The dΔ*12*, dΔ*23*, and dΔ*13* mutant strains were mutated at EPS gene clusters 1 and 2, EPS gene clusters 2 and 3, and EPS gene clusters 1 and 3, respectively. Thus, it was observed that these mutants produced EPS_III_, EPS_I_, and EPS_II_, respectively (27). In the beginning of biofilm formation, 10 nM rFlaE or rFlaF protein was included. The *P* values for the comparison with a control (no addition) are indicated (*, 0.005 ≤ *P < *0.05; **, *P < *0.005). (B) High copy numbers of *flaE* or *flaF* in various EPS mutants. The standard biofilm assay was performed using the wild type carrying pRK415, the dΔ*12* mutant carrying pRK415, the dΔ*12* mutant carrying pRK415-*flaE*, the dΔ*12* mutant carrying pRK415-*flaF*, the dΔ*23* mutant carrying pRK415, the dΔ*23* mutant carrying pRK415-*flaE*, the dΔ*23* mutant carrying pRK415-*flaF*, the dΔ*13* mutant carrying pRK415, the dΔ*13* mutant carrying pRK415-*flaE*, and the dΔ*13* mutant carrying pRK415-*flaF.* The *P* values for the comparison with a control (vector only) are indicated (*, 0.005 ≤ *P < *0.05; **, *P < *0.005). (C and D) EPS fractions were prepared from the wild type and the dΔ*12*, dΔ*23*, and dΔ*13* mutants and then dissolved in PBS. Five micrograms of each EPS fraction was added to the biofilm assays using either the *flaE*::*nptI* (C) or Δ*flaF* (D) mutant. As negative controls, both PBS and the final fractionation of the EPS extraction procedure applied to the tΔ123 triple mutant were included in this assay. The *P* values for the comparison with controls (PBS) are indicated (*, 0.005 ≤ *P < *0.05; **, *P < *0.005).

Since the amino acid sequences of FlaE and FlaF show 53.2% identity, it is plausible to speculate that each FHP might interact with a different kind of EPS. To dissect the specific interaction between FHPs and EPS, each FHP was administered to various EPS mutants, including CBdΔ*12*, CBdΔ*13*, and CBdΔ*23* strains, which produce EPS_III_, EPS_II_, and EPS_I_, respectively (29). The degrees of formation of biofilms by CBdΔ*12* and CBdΔ*23* were increased by the addition of rFlaE, but those by CBdΔ*13* were increased by the addition of rFlaF only (Fig. 7A). These observations showing the specific interaction of FlaE with EPS_I_/EPS_III_ and that of FlaF with EPS_II_ were confirmed by using EPS mutant strains carrying multiple copies of *flaE* or *flaF*. For this purpose, strains of CBdΔ*12*, CBdΔ*23*, and CBdΔ*13* carrying plasmid pRK415, pRK415-*flaE*, or pRK415-*flaF* were prepared. Their abilities to form biofilms were compared with that of the wild type carrying pRK415 (Fig. 7B). As shown in Fig. 7A, addition of high copy numbers of the *flaE* gene to the strains producing EPS_I_ or EPS_III_ (CBdΔ*23* and CBdΔ*12*, respectively) resulted in increased biofilm formation, and addition of high copy numbers of the *flaF* gene to the EPS_II_-producing strain (CBdΔ*13*) resulted in increased biofilm formation.

Another biofilm assay was performed using the *flaE*::*nptI* and Δ*flaF* mutants with exogenous addition of various kinds of EPS which have been fractionated from the wild type, CBdΔ*12*, CBdΔ*23*, or CBdΔ*13*. The degree of biofilm formation by the *flaE*::*nptI* mutant (producing FlaF only) was increased only with the addition of EPS extracted from CBdΔ*13* (Fig. 7C), but the degree of biofilm formation by the Δ*flaF* mutant (producing FlaE only) was increased with addition of EPS extracted from CBdΔ*12* and CBdΔ*23* (Fig. 7D). These results consistently supported the above-described observations showing the specific interactions of FlaE and FlaF with EPS_I_/EPS_III_ and EPS_II_, respectively.

**FIG 8:**
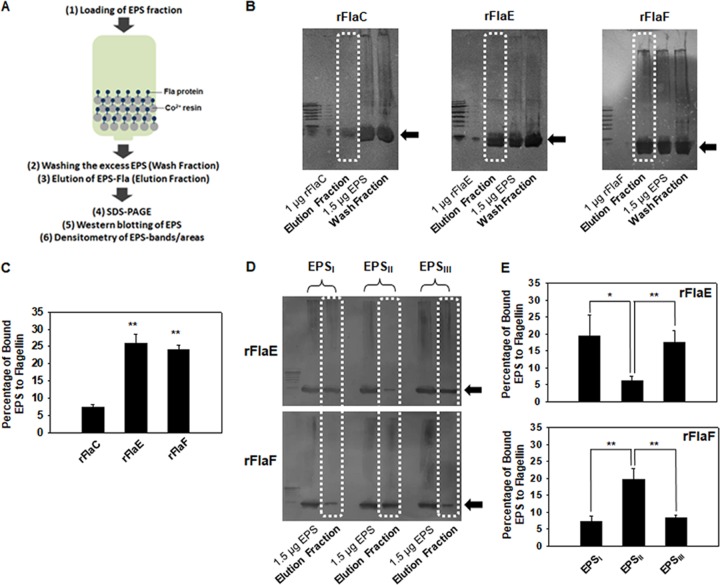
Specific interactions of FlaE/F with EPS_I_/EPS_II_/EPS_III_. (A) Flow chart for the pulldown assay using EPS. To confirm the interactions between two FHPs and EPSs, a pulldown assay was performed as described in Materials and Methods. EPS were applied to the resins bound by His-tagged FlaE, -F, or -C. After washing the excess EPSs, the bound EPS were retrieved using elution buffer. Each fraction (elution fraction and wash fraction) was subjected to SDS-PAGE and subsequent Western blotting using anti-EPS polyclonal antibodies. Intensity of antibody-reacting areas/bands was quantified by densitometry, and the percentage of bound EPS was calculated. For this quantitative comparison, EPS of 1.5 μg of Glc-eq. was also included in each Western blot. (B and C) Pulldown assay using EPS from the wild type (EPS_WT_). EPS fractions which had been tightly bound to rFlaE, rFlaF, or rFlaC were eluted and then visualized by Western blotting. In addition to the elution fractions, the initially added EPS preparation and the washed fractions were also included. A representative blot from three independent assays is presented in panel B. The intensity of immunoreactive areas on the lanes containing elution fractions (as indicated with dashed boxes) were estimated and compared in a bar graph (C). The *P* values for the comparison with rFlaC are indicated (**, *P < *0.005). (D and E) Pulldown assay using EPS_I_, EPS_II_, or EPS_III_. To verify the specific interactions between two FHPs and three EPS, pulldown assays were performed by applying EPS_I_, EPS_II_, or EPS_III_ to the column containing resins coated with either rFlaE or rFlaF. A representative blot from five independent assays for each protein is presented in panel D. The quantification procedure for the bound EPS was followed as described above. The *P* values for the comparison among different EPS are indicated (*, 0.005 ≤ *P < *0.05; **, *P < *0.005).

To test whether FHPs directly interact with EPS, a pulldown assay, in which a recombinant protein on the Co^2+^ resin was exposed to polysaccharides, and a subsequent immunoreactive blotting of the resin-bound polysaccharides using the polyclonal antibodies raised against EPS were adopted (Fig. 8A). Immunoblots of the EPSs fractionated from V. vulnificus showed a broad range of their sizes, from higher molecular weights to relatively lower molecular weights (designated with arrows in Fig. 8) that appeared in concentrated forms in the prepared EPS fractions (lanes labeled “1.5 μg EPS” in Fig. 8B). The pulldown assays showed that most EPSs applied to the resins were washed away due to excess amounts of EPS added (lanes labeled “Wash Fraction” in Fig. 8B); however, the detectable amounts of bound EPS were able to be retrieved by the elution process (lanes labeled “Elution Fraction” in Fig. 8B). Both rFHPs interacted with EPS_WT_, which was extracted from wild type and thus included EPS_I_, EPS_II_, and EPS_III_, at significantly higher levels than rFlaC (Fig. 8B). Densitometric analyses of the bound EPS (boxed with dotted lines on the lanes labeled “Elution Fraction”) and the known amount of EPS (the lanes labeled “1.5 μg EPS”) revealed that the levels of EPS bound to rFlaE or rFlaF were 3.5 times higher than for EPS bound to rFlaC (Fig. 8C).

To differentiate the specificity between FHPs and EPSs, pulldown assays were performed using rFlaE and rFlaF with EPS_I_, EPS_II_, or EPS_III_. The resultant immunoblots of EPS exposed to rFlaE showed stronger interactions with EPS_I_ and EPS_III_ than with EPS_II_ (upper blot in Fig. 8D). In contrast, the EPS bound to rFlaF was revealed to be EPS_II_ (lower blot in Fig. 8D). Densitometric analyses revealed significant difference in each EPS bound to rFlaE or rFlaF, showing that 2.8 to 3.1 times more EPS_I_ and EPS_III_ were bound to rFlaE than EPS_II_ and 2.6 times more EPS_II_ was bound to rFlaE than EPS_I_ and EPS_III_ (Fig. 8E; *P* < 0.05, Student’s *t* test). Therefore, these results strongly indicated that FlaE specifically interacted with the EPS synthesized by the EPS clusters 1 and 3 and FlaF specifically interacted with the EPS synthesized by the EPS cluster 2.

### FHPs in other pathogenic *Vibrio* species.

**FIG 9:**
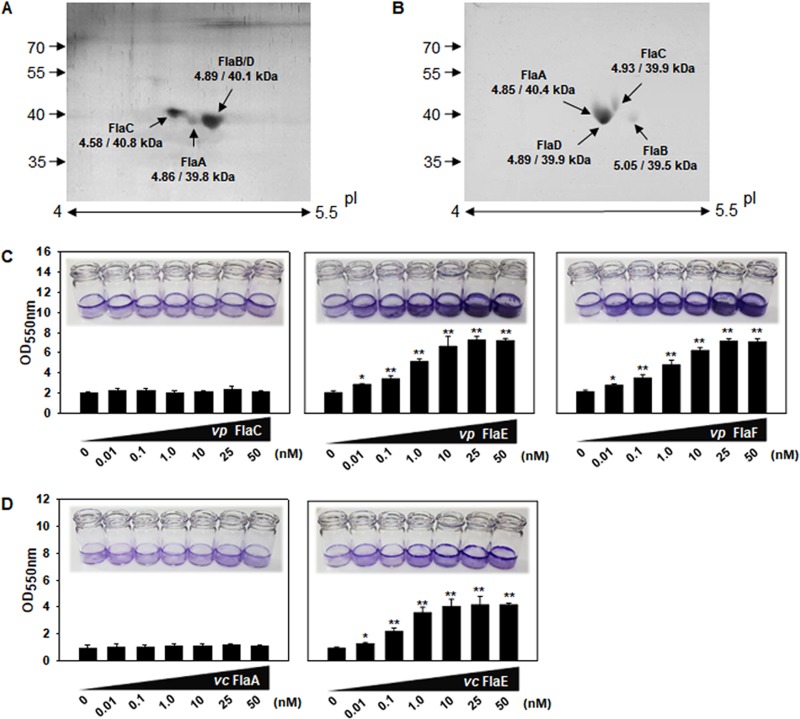
Role of FHPs in biofilm formation by V. parahaemolyticus and V. cholerae. (A and B) Composition of the flagellar filaments of V. parahaemolyticus and V. cholerae. Flagellar fractions were harvested from V. parahaemolyticus RIMD2210633 (A) and V. cholerae ATCC 14033 (B) grown in AB-fumarate or LB medium for 16 h, respectively. Fifty micrograms of flagellar preparation was subjected to 2-D gel analysis, as described for Fig. 1D. The protein spots are indicated by the corresponding annotation with the molecular weights and pIs calculated from their amino acid sequences. (C and D) Effect of exogenous addition of rFHPs on biofilm formation. Biofilm growth of V. parahaemolyticus and V. cholerae was monitored in the presence of the recombinant FHP proteins, at concentrations ranging from 0.01 to 50 nM. FHPs of V. parahaemolyticus are FlaE and FlaF (C), and the FHP of V. cholerae is FlaE (D). For comparison, biofilm assays using the same concentrations of one of the flagellin subunits (rFlaC of V. parahaemolyticus [C] and rFlaA of V. cholerae [D]) were also performed. Incubation procedures and biofilm estimation were as described for Fig. 4A. The *P* values for the comparison with a negative control (0 nM FHP added) are indicated (*, 0.005 ≤ *P < *0.05; **, *P < *0.005).

Based upon the genome sequences of other pathogenic *Vibrio* species, there are multiple ORFs encoding putative flagellin subunits. Although V. parahaemolyticus RIMD2210633 has six ORFs, only three protein spots corresponding to four flagellins, FlaA_vp_, FlaB_vp_/D_vp_, and FlaC_vp_, were present in its flagellar filament (Fig. 9A), as for V. vulnificus. V. cholerae ATCC 14033 has five ORFs; however, its filament contained four flagellins, FlaA_vc_, FlaB_vc_, FlaC_vc_, and FlaD_vc_ (Fig. 9B), as previously reported for another strain of V. cholerae (18). To examine whether the rFHPs of V. parahaemolyticus and V. cholerae could play any role in biofilm formation as shown in V. vulnificus, their rFHPs and one of the flagellin subunits were purified and subjected to the standard biofilm assays. Exogenous addition of rFHPs of V. parahaemolyticus (FlaE_vp_ [VP0791] and FlaF_vp_ [VP2261]) significantly increased biofilm formation by V. parahaemolyticus, while a recombinant flagellin of V. parahaemolyticus (FlaC_vp_ [VP0788]) did not affect biofilm formation (Fig. 9C). An approximately 3.8-fold increase in biofilm formation was observed with addition of rFHPs up to concentrations of 25 nM. Similarly, in the case of biofilm formation by V. cholerae, significant increases of biofilms were induced by exogenous addition of rFHPs of V. cholerae (FlaE_vc_ [VC_2144]) in a dose-dependent manner up to 10 nM, but no difference was observed in sizes of biofilms formed in the presence of a recombinant flagellin of V. cholerae (FlaA_vc_ [VC_2188]) (Fig. 9D).

## DISCUSSION

As shown for various *Vibrio* species, ORFs encoding flagellin-homologous proteins that are not involved in filament assembly are present in their genomes (15, 18, 19). Among the six putative flagellin ORFs in V. vulnificus, all the six ORFs were found to be well expressed (Fig. 1B and Fig. 2A). Studies on the transcription of each ORF showed that one of the ORFs encoding FHPs, *flaE*, is organized as an operon with the flagellin gene, *flaD* (Fig. 1C), which supports the occurrence of the *flaE* transcription as a polycistronic transcript containing *flaD* and *flaE*. However, only four flagellin subunits were found in its filaments (Fig. 1D). This is consistent with previous findings by 2-D gel electrophoresis and a subsequent MALDI-TOF analysis (19), although it was noted in the same report that a liquid chromatography-mass spectrometry (LC-MS) method could detect a peptide derived from FlaF. This discrepancy is speculated to be caused by coprecipitation of a minor portion of FHPs in the flagellar fraction, since FHPs are also secreted through the same secretion apparatus as for flagellar components, such as FlhA (Fig. 3A). Although FHPs pass through the same channel as the flagellin subunits, the FHPs exhibited significantly lower affinities for HAPs, such as FliD and FlgL (Fig. 3B). Since the interactions among proteins of HAPs and flagellins are important in self-assembly of flagella (10, 25, 26), it is assumed that FHPs are just excreted without being assembled into flagellar structure, as shown in Fig. 2B.

In addition, the FHPs exhibited almost no affinity for FlaB (Fig. 3B). The flagellin interaction has been shown to be mediated via formation of the complementary shapes between the upper and lower subunits in a filament (30). The axial flagellin interactions are formed between a concave surface in the domain D1 of the upper flagellin and a convex surface in domains D1/D2a of the lower flagellin. In V. vulnificus FHPs, the amino acid residues in these two surfaces are different from those of FlaA/B/C/D, which commonly contain Ser_58_, Ser_107_, and Thr_141_ in the tentative interacting surfaces. However, FHPs have different amino acids residues in the corresponding sites; FlaE has Thr_57_, Asp_106_, and Ser_140_ and FlaF has Met_58_, Ala_107_, and Ser_141_. Thus, it was speculated that no interaction of FHPs with flagellins would also facilitate the excretion of FHPs through the flagellin secretion apparatus.

In the presence of exogenously added FHPs, *Vibrio* species were shown to form increased biofilms in a dose-dependent manner (Fig. 4 and 9). In V. vulnificus, the incorporation of FHPs into the biofilm matrix was evident (Fig. 5). FHP-mediated increase in biofilm formation was driven by their interactions with the major components in the biofilm matrix, EPSs (Fig. 6 and 7). EPSs are essential in maturing biofilms formed by V. vulnificus (29). V. vulnificus has at least three EPS biosynthesis gene clusters (EPS clusters) in its genome. Each EPS cluster contains its own exporter for EPS and the ATPase-type biosynthesis protein at the third and fourth loci, suggesting that each EPS cluster is responsible for the biosynthesis of a distinct polysaccharide (29). The EPS I cluster is composed of the genes homologous to *syp* genes of a symbiotic V. fischeri (31). The EPS II cluster, which is also called the *wcr* or *brf* gene cluster (32), is found in the genomes of V. cholerae and V. parahaemolyticus as *vps* and *cps* clusters, respectively. The EPS III cluster, of which ORFs are also found in the V. fischeri genome, was shown to be involved in biosynthesis of the most critical EPSs involved in adherence to host cells, lethality of mouse model, and biofilm formation of V. vulnificus (29).

Immunoblotting analyses using two recombinant FHPs and each EPS extract revealed highly specific interactions between FHPs and EPSs; the V. vulnificus FlaE and FlaF specifically bound to EPS_I_/EPS_III_ and EPS_II_, respectively (Fig. 8D and E). It was proposed that the association of EPSs with FHPs strengthened the matrix of biofilms to enhance the ability of EPSs to mature the biofilm structure. In B. subtilis, the extracellular protein TasA was required for the structural integrity of biofilms, along with an exopolysaccharide (33). The biofilms of Azospirillum brasilense have been shown to be strengthened by the membrane-bound lectin proteins (34). The secreted mannose-binding CdrA and galactose or fucose-binding lectins (i.e., LecA or LecB, respectively) were also found to be involved in biofilm formation by P. aeruginosa (35, 36).

LecA of P. aeruginosa harbors amino acid residues of His_50_, Gln_53_, Asp_100_, and Asn_107_, which are important in hydrogen bonding with galactose (37). However, it was shown that the Ser_22_, Ser_23_, Asp_96_, Asp_99_, Asp_101_, and Gly_114_ residues of LecB are involved in formation of hydrogen bonds with fucose when its Asp_104_ coordinates two calcium ions (38). In mannose-specific FimH of E. coli, Asp_54_, Gln_133_, Asn_135_, and Asp_140_ residues are important for hydrogen bond interaction with mannose (39). A carbohydrate-interacting domain in the glucan-binding protein of Streptococcus mutans contains the carboxyl-terminal repeats similar to those that make up the glucan-binding domain of the glucosyltransferase (40). Unlike several known carbohydrate binding motifs elucidated in other bacterial systems, the *Vibrio* FHPs do not exhibit the putative carbohydrate binding motif. However, alignment of amino acids of various FHPs showed some conservation of amino acid residues, which are not found in flagellins. This needs to be further studied in future studies by using various recombinant FHPs with mutagenized amino acids, in order to show the novel mode of carbohydrate-binding by FHPs.

In conclusion, FHPs in V. vulnificus do not participate in construction of flagellar filaments but are excreted to extracellular milieu, where they are involved in strengthening the EPS-enriched biofilm matrix. In addition, these observations are not limited to V. vulnificus: other pathogenic *Vibrio* species also exhibited the presence of FHPs that are involved in biofilm enhancement, which, in turn, may facilitate increased pathogenicity of these bacterial species.

## MATERIALS AND METHODS

### Bacterial strains and culture conditions.

The strains and plasmids used in this study are listed in Table S1. E. coli organisms used for plasmid DNA preparation and for conjugal transfer were grown in Luria-Bertani (LB) medium supplemented with appropriate antibiotics at 37°C. V. vulnificus and V. parahaemolyticus were grown at 30°C in LBS (LB medium containing NaCl at a final concentration of 2.5% [wt/vol]) or AB medium (300 mM NaCl, 50 mM MgSO_4_, 0.2% vitamin-free Casamino Acids, 10 mM potassium phosphate, 1 mM l-arginine, pH 7.5 [41]) supplemented with 1.0% [wt/vol] fumarate. V. cholerae was grown at 30°C in LB medium. Antibiotics were used at the following concentrations: for E. coli, ampicillin at 100 μg/ml, chloramphenicol at 20 μg/ml, kanamycin at 100 μg/ml, and tetracycline at 15 μg/ml, and for V. vulnificus, chloramphenicol at 4 μg/ml, kanamycin at 300 μg/ml, and tetracycline at 3 μg/ml.

### Construction of *luxAB*-based transcriptional fusions.

The genome database of V. vulnificus strain MO6-24/O (GenBank accession no. NC_014965.1]) shows that the putative promoters are discernible in the upstream regions of *flaA* (VVMO6_00809), *flaB* (VVMO6_00808), *flaC* (VVMO6_02255), *flaD* (VVMO6_02252), and *flaF* (VVMO6_00807). The DNA fragments including the intergenic spaces between *flaA* and *flaB*, *flaB* and *flaF*, *flaC* (VVMO6_02255) and its upstream ORF (*flgL* [VVMO6_02256]), *flaD* and *flaC*, and *flaF* and its upstream ORF (an iron-dependent peroxidase [VVMO6_00806]) were amplified using the primer sets listed in Table S2. A DNA fragment containing *flaA* promoter region, ranging from −331 to +115 (relative to TIS for *flaA*), was amplified using flaA_p-F and flaA_p-R. A DNA fragment containing the *flaB* promoter region, ranging from −351 to +126 (relative to TIS for *flaB*), was amplified using flaB_p-F and flaB_p-R. A DNA fragment containing the *flaC* promoter region, ranging from −381 to +160 (relative to TIS for *flaC*), was amplified using flaC_p-F and flaC_p-R. A DNA fragment containing the *flaD* promoter region, ranging from −584 to +118 (relative to TIS for *flaD*), was amplified using flaD_p-F and flaD_p-R. A DNA fragment containing the *flaF* promoter region, ranging from −338 to +67 (relative to TIS for *flaF*), was amplified using flaF_p-F and flaF_p-R. In the case of the upstream region of *flaE*, a DNA fragment, ranging from −329 to +38 (relative to TIS for *flaE*), was amplified, although there was no apparent promoter in this region (as explained in Results, the *flaE* ORF shares the *flaD* promoter). Amplified DNA fragments were digested with KpnI and XbaI and inserted into pHK0011 containing promoterless *luxAB* (42). The resultant plasmids were mobilized into V. vulnificus by conjugation. The light produced by exconjugant V. vulnificus harboring the plasmid was measured in the presence of 0.006% (vol/vol) *n*-decyl aldehyde using a luminometer (TD-20/20; Turner Designs). Specific bioluminescence was calculated by normalizing the relative light units (RLU) with respect to cell mass (OD_595_) as described previously (42).

10.1128/mBio.01793-19.5 TABLE S2 Bacterial strains and plasmids used in this study. Download Table S2, PDF file, 0.3 MB. Copyright © 2019 Jung et al. 2019 Jung et al. This content is distributed under the terms of the Creative Commons Attribution 4.0 International license.

### RT-PCR.

Total RNA was extracted from wild-type V. vulnificus cells freshly grown in LBS, and their cDNA was prepared by performing reverse transcriptase (Moloney murine leukemia virus [M-MLV] RT; TaKaRa) reactions using 2.5 μg of the total RNA and the random 6-mer primers (TaKaRa). Then the resulting cDNA was subjected to PCR using three sets of primers (flaD-F and flaD-R, flaE-F and flaE-R, and flaD-F and flaE-R flaE-R). The expected sizes of each PCR product were 184, 86, and 436 bp when genomic DNA (gDNA) of V. vulnificus was used as a template. Absence of gDNA contamination in the RNA sample was confirmed by PCR of the RNA sample without RT reaction (43). The primers used in this assay are listed in Table S2.

### Purification of flagellar filaments and 2-dimensional gel electrophoresis.

Freshly grown cells of V. vulnificus, V. parahaemolyticus, and V. cholerae were harvested and resuspended with 30 ml of phosphate-buffered saline (PBS; 100 mM NaCl, 20 mM sodium phosphate, pH 7.5) and then blended at a low blender setting for 1.5 min. Bacterial cells were removed by centrifugation at 10,000 rpm for 10 min, and the supernatants were subjected to 0.4 g/ml of CsCl_2_ density gradient ultracentrifugation at 100,000 × *g* and 25°C for 18 h. A flagellum band was isolated using a syringe and dialyzed against distilled water for 12 h (18).

Protein concentrations in the flagellar fractions were determined using a Bradford protein assay kit (Bio-Rad), and 50 μg of protein was added to rehydration buffer {8 M urea, 2% [vol/vol] 3-[(3-cholamidopropyl) dimethylammonio]-1-propanesulfonate, 18 mM dithiothreitol [DTT], 0.5% [vol/vol] IPG buffer (GE Healthcare), and 0.002% [wt/vol] bromophenol blue]. Immobiline DryStrip (pH 4 to 7, 13 cm; Amersham Biosciences) with the sample mixture was subjected to the isoelectric focusing, which was carried out on Ettan IPGphor II^TM^ (Amersham Biosciences) under the following conditions: rehydration for 16 h, 500 V for 1 h, 1,000 V for 1 h, and 8,000 V for 3.5 h. The strips were then equilibrated in SDS equilibration buffer (50 mM Tris-HCl [pH 8.8], 6 M urea, 30% [vol/vol] glycerol, 2% [wt/vol] SDS, and 0.002% [wt/vol] bromophenol blue) containing 6% (wt/vol) DTT. Equilibrated strips were rinsed with distilled water and then transferred onto 12% polyacrylamide gels. Electrophoresis was performed at 40 mA per gel. Following electrophoresis, protein spots were detected using a Silver Stain Plus kit (Bio-Rad). The protein spots derived from V. vulnificus flagellum were excised and digested with trypsin for further analysis using a MALDI-TOF Voyager DE-STR biospectrometry workstation (Applied Biosystems). The resulting trypsin peptide profiles were used to search the Swiss-Prot protein database primarily by the MS-Fit search engine.

### Construction of deletion mutants.

Mutant strains of V. vulnificus were constructed by deleting the corresponding genes or inserting a kanamycin resistance gene (*nptI* derived from pUC4K [Pharmacia Biotech]) into the target genes using suicide vector pDM4 (44), as previously described (45). All the plasmids and information for the primers used in this study are listed in Tables S1 and S2, respectively. E. coli SM10λ*pir* carrying mutated genes was conjugated with V. vulnificus MO6-24/O and the exconjugants were selected on thiosulfate citrate bile sucrose medium supplemented with 3 μg/ml of chloramphenicol. Colonies with characteristics indicating a double homologous recombination event were isolated (resistance to 5% [wt/vol] sucrose, sensitivity to chloramphenicol, and/or resistance to kanamycin). Mutation in the target genes of candidate colonies was confirmed by PCR with specific primer sets and by complementation with broad-host-range vector pRK415 harboring the intact target gene (Table S2). The detailed procedures are provided in the supplemental material.

### Western blotting.

Cell lysates of wild-type and mutant strains of V. vulnificus were prepared, and 60-μg quantities of protein extracts were used for sodium dodecyl sulfate-polyacrylamide gel electrophoresis (SDS-PAGE). Protein pools in the supernatants of the same cultures used for collecting the cell lysates were precipitated by treatment with 10% (vol/vol) trichloroacetic acid, and 5-μg quantities of proteins in the supernatant concentrates were fractionated by SDS-PAGE. Blotted Hybond polyvinylidene difluoride (PVDF) membranes were incubated with polyclonal antibodies raised against various recombinant proteins, including flagellins, FHPs, IIA^Glc^, or OmpU (1:5,000 [vol/vol]), followed by alkaline phosphatase-conjugated rabbit anti-rat IgG (1:5,000 [vol/vol]). Immunoreactive bands were visualized using the nitroblue tetrazolium (NBT)–5-bromo-4-chloro-3-indolylphosphate (BCIP) system (Promega). The intensities of the protein bands were quantified using a densitometer (Bio-Rad; Gel Doc 2000 system).

### Observation of motility and flagellum.

The effect of mutations on swimming motility was assessed by examining bacterial motility on AB-fumarate medium containing 0.3% (wt/vol) agar. To document swimming motility, plates were spot inoculated with 1 μl of cell cultures (OD_595_ of 1.0) and incubated at 30°C for 8 h. As a negative control, a nonmotile *flgK* mutant was included.

To examine the presence or absence of flagella and the measure the lengths of flagella, electron microscopy was used. From the fresh cultures of various V. vulnificus cells at the mid-log phase (OD_595_ of ∼1.0) in AB-fumarate medium, bacterial cells were harvested and resuspended in 500 μl of PBS. The carbon-coated, copper mesh grids, which had been pretreated with 0.1% (wt/vol) bovine serum albumin (BSA), were incubated with cell resuspension for 2 min. The grids were rinsed and floated on 100 μl of 1% (wt/vol) uranyl acetate solution (46). The sample preparations were examined using a Philips CM100 transmission electron microscope (Philips Corporation) operating at 100 kV, and the images were captured using iTEM acquisition and analysis software (Olympus Soft Imaging Solutions).

### Cell adherence assay.

Assays for adherence of V. vulnificus were performed with INT-407 cells (ATCC CCL-6) derived from human intestinal epithelium (47). Each well on 24-well culture plates was seeded with about 1 × 10^5^ INT-407 cells and grown overnight at 37°C in the presence of 5% CO_2_. The cells for the assay were prepared by removing the medium, washing them twice with Hanks’ balanced salt solution (Sigma-Aldrich), and then adding 1 ml of serum-free minimal essential medium with Earle’s salt (Sigma-Aldrich). Cell monolayers were then inoculated in quadruplicate with 100 μl of the diluted bacterial cells to give an MOI of ca. 10 and were incubated for 15 min. The monolayer was washed three times with prewarmed PBS to remove nonadherent bacterial cells. After the last wash, the INT-407 cells were treated with 0.1% Triton X-100 for 15 min. The bacterial cells were recovered and plated on LBS agar plates. The degree of cytoadherence was presented as the percentage of retrieved bacterial cells out of the initial inoculated bacteria, as previously described (27).

### Assay using the BACTH system.

The bacterial two-hybrid (BACTH) system used in this study consists of two plasmids, pUT18c and pKT25 (Euromedex), into which the ORF of the flagellar component (*flaB*, *fliD*, or *flgL*) or FHP (*flaE* or *flaF*) was inserted. A combination of two ORFs in each plasmid were cotransformed into E. coli BTH101, and each transformant was spotted onto an LB–X-Gal (bromo-4-chloro-3-indolyl-β-d-galactopyranoside) (40 μg/ml) agar plate containing ampicillin (100 μg/ml), kanamycin (50 μg/ml), and isopropyl-β-d-thiogalactopyranoside (IPTG; 0.5 mM) to observe the color development due to the activity of β-galactosidase. To quantify the β-galactosidase activity, enzymatic reactions using ONPG (*O*-nitrophenol-β-galactoside; 4 mg/ml) were performed. Cells grown in LB broth containing ampicillin (100 μg/ml), kanamycin (50 μg/ml), and IPTG (0.5 mM) were resuspended in Z buffer (60 mM Na_2_HPO_4_, 40 mM NaH_2_PO_4_, 10 mM KCl, 1 mM MgSO_4_, and 50 mM mercaptoethanol) and treated with 0.1% (wt/vol) SDS and chloroform to prepare crude cell lysates. After addition of ONPG (670 μg/ml), the reactions were stopped by addition of Na_2_CO_3_ solution. Optical density at 420 nm was measured for each reaction mixture, and the Miller units were calculated as follows: U = (OD_420_ × 1,000)/(time × cell culture volume × OD_600_) (48). The positive control containing pUT18c-*zip*/pKT25-*zip* and the negative control containing pUT18c/pKT25, which were provided in a commercial kit, were included.

### Cloning and purification of recombinant proteins.

To amplify DNA fragments containing the complete ORFs of the *flaA*, -*B*, -*C*, -*E*, and -*F* genes of V. vulnificus, the *flaC*, -*E*, and -*F* genes of V. parahaemolyticus, and the *flaA* and -*E* genes of V. cholerae, specific primer sets were used (Table S2). Each PCR product was cloned into pQE30 and transformed into E. coli JM109. Overexpressed recombinant proteins in the presence of 1.0 mM IPTG were purified using a nickel-nitrilotriacetic acid affinity column, as per the manufacturer’s instructions (Qiagen). Purified recombinant proteins were verified by SDS-PAGE prior to addition to the biofilm formation and EPS interaction assays. Purified recombinant proteins of V. vulnificus were also used to raise the polyclonal antibodies by treating 6-week-old female Sprague-Dawley rats with 50-μg quantities of flagellins or FHPs (the animals received humane care in accordance with our institutional guidelines and the legal requirements [IACUCSGU2019_01]).

### Biofilm formation assay.

Overnight cultures of V. vulnificus and V. parahaemolyticus were inoculated into AB-fumarate broth, and overnight cultures of V. cholerae were seeded into LB broth in borosilicate tubes. After static incubation at 30°C for 48 h, the planktonic phase in the borosilicate was removed and its cell density was measured by spectrometric reading at OD_595_. The remaining biofilms were washed with PBS and stained with 1.0% crystal violet to estimate the degree of biofilm formation. The stained biofilms were washed with distilled water, air dried, and resuspended in 100% ethanol (49). Crystal violet in stained biofilms was quantified by spectrometric reading at OD_550_.

### Measurement of rFlaC and rFHPs in biofilm matrix.

rFlaC, rFlaE, and rFlaF were labeled with a fluorescent probe, Alexa Fluor 555, using a microscale protein labeling kit (Molecular Probes). To determine the degree of labeling, the conjugate samples were subjected to absorbance analysis at 280 nm and 555 nm, as suggested in the manufacturer’s manual. The three recombinant proteins showed similar degrees of labeling, ranging from 80% to 87%, when 50- to 150-μg quantities of recombinant proteins were conjugated for 15 min. At the beginning of biofilm incubation, a 10 nM concentration of each labeled protein was added to AB-fumarate broth inoculated with the Δ*flaEF*. Biofilms formed on borosilicate tubes were washed twice with PBS and resuspended in a solution of 40 mM sodium acetate (pH 4.0). The amounts of fluorescence in biofilm resuspension were quantified by spectrometric reading at 555 nm. For confocal microscopic observation of the fluorescence-labeled proteins in biofilms, the wild-type and Δ*flaEF* strains of V. vulnificus harboring pRK415-*gfp* were inoculated to AB-fumarate containing tetracycline (3 μg/ml) and IPTG (1.0 mM) in coverslip-bottomed dishes (SPL). Formed biofilms on the coverslip were washed twice with PBS and covered with emulsion oil (Merck). The *gfp*-tagged biofilms and the Alexa Fluor 555-labeled proteins incorporated into EPM were observed using a confocal microscope (DMB4000B; Leica) at excitation and emission wavelengths of 408 and 509 nm and excitation and emission wavelengths of 555 and 565 nm, respectively. The images were processed using LAS AF software.

### Extraction and analysis of EPS.

About 5 × 10^10^ V. vulnificus cells grown on AB-fumarate agar plates were suspended in 10 ml of PBS and vigorously shaken (200 rpm) for 1 h to elute loosely associated extracellular matrix. After the removal of cells and debris, the supernatants were treated with RNase A (50 μg/ml) and DNase I (50 μg/ml) at 37°C for 8 h in the presence of 10 mM MgCl_2_. Proteinase K (200 μg/ml) was subsequently added to the reaction mixtures and incubated at 37°C for 17 h. The remaining polysaccharide fractions were extracted twice with phenol-chloroform, and then the extracts were treated with 0.3 M sodium acetate (NaOAc). EPS were precipitated by adding 2.5× volumes of ethanol. After a washing with 70% (vol/vol) ethanol, EPS pellets were resuspended in distilled water and completely dissolved by adjusting the pH to be neutral using HCl. Aliquots of EPS fractions were run on a 5% (wt/vol) stacking polyacrylamide gel and stained with Stains-All (Sigma) (50). The carbohydrate contents in each EPS fraction were estimated by the phenol-sulfuric acid method (51) using glucose as a carbohydrate standard. Thus, the amounts or concentrations of each EPS were presented as the glucose equivalents (Glc-eq.). To obtain the EPS-specific polyclonal antibodies, 6-week-old female Sprague-Dawley rats were immunized intraperitoneally with 200 μg of Glc-eq. of EPS. The animals were boosted twice at 2-week intervals with the same amount of antigens. Four days after the third immunization, blood samples of rats were pooled and used for further experiments as polyclonal antibodies against EPS. The animals received humane care in accordance with our institutional guidelines and the legal requirements (IACUCSGU2019_01). The specificity of the resulting antibodies was confirmed by immunoblotting the EPS extract and other extracellular polysaccharides, such as LPS or CPS (Fig. S3).

10.1128/mBio.01793-19.3 FIG S3 Specificity of anti-EPS polyclonal antibodies. Three kinds of extracellular polysaccharides produced by V. vulnificus, LPS, EPS, and CPS, were extracted as described previously (K. J. Lee, J. A. Kim, W. Hwang, S. J. Park, and K. H. Lee, Mol Microbiol 90:841–857, 2013, https://doi.org/10.1111/mmi.12401; H.-S. Kim, M.-A. Lee, S.-J. Chun, S.-J. Park, K.-H. Lee, Mol Microbiol 63:559–574, 2007, https://doi.org/10.1111/j.1365-2958.2006.05527.x) or in Materials and Methods. Five micrograms of each carbohydrate (equivalent to the amounts of 3-deoxy-**d*-*manno-octulosonic acid [KDO], glucose [Glc], and galacturonic acid [GalA] for LPS, EPS, and CPS, respectively [as previously described in the following references: K. J. Lee, J. A. Kim, W. Hwang, S. J. Park, and K. H. Lee, Mol Microbiol 90:841–857, 2013, https://doi.org/10.1111/mmi.12401; H.-S. Kim, M.-A. Lee, S.-J. Chun, S.-J. Park, K.-H. Lee, Mol Microbiol 63:559–574, 2007, https://doi.org/10.1111/j.1365-2958.2006.05527.x; and H.-S. Kim, S.-J. Park, K.-H. Lee, Mol Microbiol 74:436–453, 2009, https://doi.org/10.1111/j.1365-2958.2009.06875.x]) were run on 5% (vol/vol) stacking polyacrylamide gel and stained with Stains-All (A). The same amounts of each carbohydrate in a stacking gel were subjected to Western blotting using anti-EPS polyclonal antibodies (B). Please note that the antibodies used in this study reacted only with EPS. Download FIG S3, TIF file, 0.9 MB. Copyright © 2019 Jung et al. 2019 Jung et al. This content is distributed under the terms of the Creative Commons Attribution 4.0 International license.

### EPS-protein binding assay.

Various EPS fractions, EPS_wt_, EPS_I_, EPS_II_, EPS_III_, and EPS_Δt123_, corresponding to 20 μg of Glc-eq. were passed through columns containing resins bound by 10 μg of His-tagged recombinant proteins (rFlaE, rFlaF, or rFlaC). After three washings with buffer (50 mM NaH_2_PO_4_, 300 mM NaCl, 20 mM imidazole, pH 8.0), EPSs associated with recombinant proteins were retrieved by treating resins with an elution buffer (50 mM NaH_2_PO_4_, 300 mM NaCl, 250 mM imidazole [pH 8.0]). Aliquots of the elution fraction and wash fraction were subjected to SDS-PAGE and electrotransferred onto a PVDF membrane. For comparison, a known amount of EPS (1.5 μg of Glc-eq.) was also included in each Western blot. After blocking with 5% (wt/vol) skim milk, the membrane was sequentially incubated with EPS-specific polyclonal antibodies and alkaline phosphatase-conjugated anti-rat IgG. Immunoreactive EPS bands were visualized using the NBT-BCIP system, their intensities were quantified via densitometry, and then the percentage of bound EPS in the elution fraction was calculated by comparing the intensities of antibody-reacting areas/bands derived from the known amount of EPS.

10.1128/mBio.01793-19.6 TABLE S3 Oligonucleotides used in this study. Download Table S3, PDF file, 0.3 MB. Copyright © 2019 Jung et al. 2019 Jung et al. This content is distributed under the terms of the Creative Commons Attribution 4.0 International license.

### Statistical analyses.

Results were expressed as means ± standard deviations from at least three independent experiments. Statistical analysis was performed using Student's *t* test (SYSTAT program; SigmaPlot version 9, Systat Software Inc.). *P* values are presented in the corresponding figures, or their significance is indicated by asterisks (*, 0.005 ≤ *P* < 0.05; **, *P* < 0.005).
