# Supplementary material for: Role of Flagellin-Homologous Proteins in Biofilm Formation by Pathogenic Vibrio Species
Source: mBio. 2019 Aug 13;10(4):e01793-19. doi: 10.1128/mBio.01793-19 (PMC6692518; doi:10.1128/mBio.01793-19)
Supplement: TABLE S1 [file mBio.01793-19-st001.pdf]

Table S1. Gene numbers and annotation of ORFs in the Regions I and II of flagellar assembly in *V. vulnificus* MO6-24/O

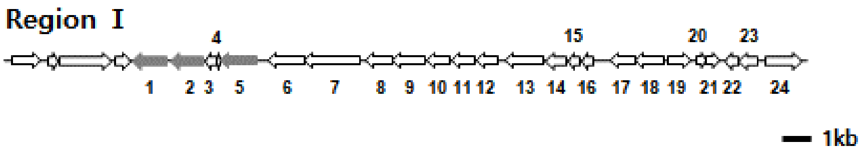

No. Gene No. [1]	Functions/Structures [2]	Gene name
1. VVMO6_02251	flagellin-like protein	*flaE*
2. VVMO6_02252	flagellin	*flaD*
3. VVMO6_02253	hypothetical protein	-
4. VVMO6_02254	hypothetical protein	-
5. VVMO6_02255	flagellin	*flaC*
6. VVMO6_02256	flagellar hook-associated protein (HAP3)	*flgL*
7. VVMO6_02257	flagellar hook-associated protein (HAP1)	*flgK*
8. VVMO6_02258	peptidoglycan hydrolase	*flgJ*
9. VVMO6_02259	P-ring protein	*flgI*
10. VVMO6_02260	L-ring protein	*flgH*
11. VVMO6_02261	basal-body rod protein	*flgG*
12. VVMO6_02262	basal-body rod protein	*flgF*
13. VVMO6_02263	hook protein	*flgE*
14. VVMO6_02264	flagellar hook-scaffolding protein [3]	*flgD*
15. VVMO6_02265	basal-body rod protein	*flgC*
16. VVMO6_02266	basal-body rod protein	*flgB*
17. VVMO6_02267	chemotaxis protein methyltransferase	*cheR*
18. VVMO6_02268	CheW-like coupling protein [4]	*cheV*
19. VVMO6_02269	basal-body P-ring formation protein	*flgA*
20. VVMO6_02270	anti-σ^{28} factor	*flgM*
21. VVMO6_02271	FlgKL-specific chaperone [5]	*flgN*
22. VVMO6_02272	flagellar lipoprotein [6]	*flgP*
23. VVMO6_02273	flagellar membrane protein [6]	*flgO*
24. VVMO6_02274	basal-body associated protein [7]	*flgT*

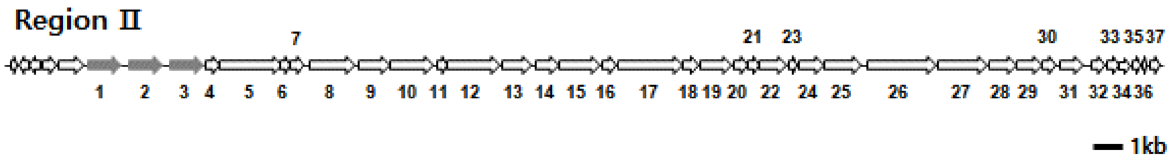

No. Gene No. (1)	Functions/Structures (2)	Gene name
1. VVMO6_00807	flagellin-like protein	*flaF*
2. VVMO6_00808	flagellin	*flaB*
3. VVMO6_00809	flagellin	*flaA*
4. VVMO6_00810	truncated flagellin	*flaG*
5. VVMO6_00811	flagellar hook-associated protein (HAP2)	*fliD*
6. VVMO6_00812	potential chaperone	*flaI*
7. VVMO6_00813	flagellin-specific chaperone	*flaJ* (*fliS*)
8. VVMO6_00814	flagellar regulatory protein	*flaK* (*flrA*)
9. VVMO6_00815	flagellar sensor histidine kinase	*flaL* (*flrB*)
10. VVMO6_00816	flagellar regulatory protein	*flaM* (*flrC*)
11. VVMO6_00817	hook-basal body complex protein	*fliE*
12. VVMO6_00818	M-ring protein	*fliF*
13. VVMO6_00819	motor switch protein	*fliG*
14. VVMO6_00820	flagellar export/assembly apparatus (8)	*fliH*
15. VVMO6_00821	flagellar export/assembly ATPase (8)	*fliI*
16. VVMO6_00822	flagellar export/assembly apparatus (9)	*fliJ*
17. VVMO6_00823	hook-length control protein	*fliK*
18. VVMO6_00824	stator-associated protein (10)	*fliL*
19. VVMO6_00825	motor switch protein	*fliM*
20. VVMO6_00826	motor switch protein	*fliN*
21. VVMO6_00827	flagellar export/assembly apparatus	*fliO*
22. VVMO6_00828	flagellar export/assembly apparatus	*fliP*
23. VVMO6_00829	flagellar export/assembly apparatus	*fliQ*
24. VVMO6_00830	flagellar export/assembly apparatus	*fliR*
25. VVMO6_00831	flagellum-specific transport apparatus (11)	*flhB*
26. VVMO6_00832	flagellum-specific transport apparatus (11)	*flhA*
27. VVMO6_00833	flagellar biosynthesis protein	*flhF*

28. VVMO6_00834	flagellar biosynthesis regulator	*flhG*
29. VVMO6_00835	sigma F, σ^{28}	*fliA*
30. VVMO6_00836	signal transduction to motor (4)	*cheY*
31. VVMO6_00837	phosphatase dephosphorylating CheY (4)	*cheZ*
32. VVMO6_00838	histidine kinase phosphorylating CheY/B (4)	*cheA*
33. VVMO6_00839	chemotaxis protein methylesterase (4)	*cheB*
34. VVMO6_00840	SOJ-like ATPase	-
35. VVMO6_00841	hypothetical protein with CheW-domain	-
36. VVMO6_00842	histidine kinase-coupling protein (4)	*cheW*
37. VVMO6_00843	hypothetical protein with ATPase activity	-

(1) GenBank under accession no. CP002469.1 and CP002470.1 (Park JH, Cho YJ, Chun J, Seok YJ, Lee JK, Kim KS, Lee KH, Park SJ, Choi SH. 2011. Complete genome sequence of *Vibrio vulnificus* MO6-24/O. J Bacteriol 193:2062-2063)

(2) Functions and/or structures of some gene products were derived from the information of the *V. parahaemolyticus* homologs in the GenBank under accession no. of BA000031.2 and BA000032.2 (Makino K, Oshima K, Kurokawa K, Yokoyama K, Uda T, Tagomori K, Iijima Y, Najima M, Nakano M, Yamashita A, Kubota Y, Kimura S, Yasunaga T, Honda T, Shinagawa H, Hattori M, Iida T. 2003. Genome sequence of *Vibrio parahaemolyticus*: A pathogenic mechanism distinct from that of *V. chorelae*. Lancet 361:743-749)

(3) Ohnishi K, Ohto Y, Aizawa S, Macnab RM, Iino T. 1994. FlgD is a scaffolding protein needed for flagellar hook assembly in *Salmonella typhimurium*. J Bacteriol 176:2272-2281

(4) Ortega A, Zhulin IB, Krell T. 2017. Sensory repertoire of bacterial chemoreceptors. Microbiol Mol Biol Rev 81: e00033-17

(5) Fraser GM, Bennett JC, Hughes C. 1999. Substrate-specific binding of hook-associated proteins by FlgN and FliT, putative chaperones for flagellum assembly. Mol Microbiol 32:569-580

(6) Martinez RM, Dharmasena MN, Kirn TJ, Taylor RK. 2009. Characterization of two outer membrane proteins, FlgO and FlgT, that influence *Vibrio cholerae* motility. J Bacteriol 191:5669-5679

(7) Martinez RM, Jude BA, Kirn TJ, Skorupski K, Taylor RK. 2010. Role of FlgT in anchoring the flagellum of *Vibrio cholerae*. J Bacteriol 192:2085-2092

(8) Minamino T, Yoshimura SD, Morimoto YV, Gonzalez-Pedrajo B, Kami-Ike N, Namba K. 2009. Roles of the extreme N-terminal region of FliH for efficient localization of the FliH-FliI complex to the bacterial flagellar type III export apparatus. Mol Microbiol 74:1471-1483

(9) Ibuki T, Uchida Y, Hironaka Y, Namba K, Imada K, Minamino T. 2013. Interaction between FliJ and FlhA, components of the bacterial flagellar type III export apparatus. J Bacteriol 195:466-473

(10) Takekawa N, Isumi M, Terashima H, Zhu S, Nishino Y, Sakura M, Kojima S, Homma M, Imada K. 2019. Structure of *Vibrio* FliL, a new stomatin-like protein that assists the bacterial flagellar motor function. mBio 10:e00292-19

(11) Minamino T, Iino T, Kutuskake K. 1994. Molecular characterization of the *Salmonella typhimurium flhB* operon and its protein products. J Bacteriol 176:7630-7637
