# Supplementary figures and images for: Role of Flagellin-Homologous Proteins in Biofilm Formation by Pathogenic Vibrio Species
Source: mBio. 2019 Aug 13;10(4):e01793-19. doi: 10.1128/mBio.01793-19 (PMC6692518; doi:10.1128/mBio.01793-19)

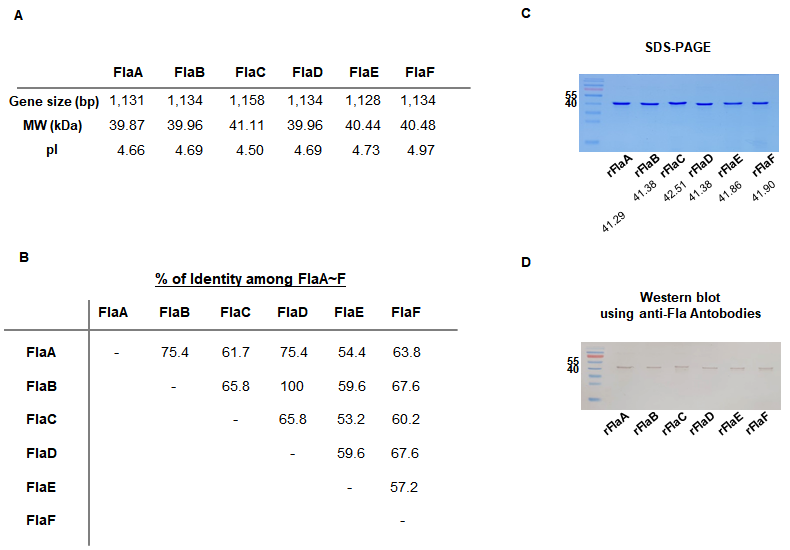

Supplement: FIG S1 [file mBio.01793-19-sf001.tif]

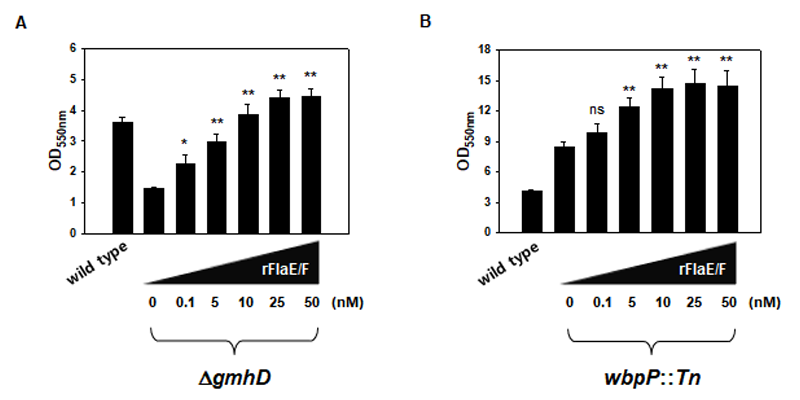

Supplement: FIG S2 [file mBio.01793-19-sf002.tif]

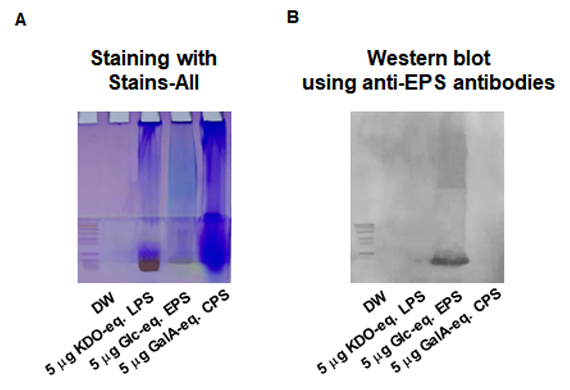

Supplement: FIG S3 [file mBio.01793-19-sf003.tif]
