# Supplementary material for: Role of Flagellin-Homologous Proteins in Biofilm Formation by Pathogenic Vibrio Species
Source: mBio. 2019 Aug 13;10(4):e01793-19. doi: 10.1128/mBio.01793-19 (PMC6692518; doi:10.1128/mBio.01793-19)
Supplement: TABLE S2 [file mBio.01793-19-st002.pdf]

Table S2. Strains and plasmids used in this study

Strain/Plasmid	Genotype	Reference [1]
V. vulnificus		
MO6-24/O	Clinical isolate	(1)
flaE::*nptI*	MO6-24/O, *flaE*, Km^R	This study
Δ*flaF*	MO6-24/O, Δ*flaF*, Km^R	This study
Δ*flaEF*	MO6-24/O, Δ*flaEF*, Km^R	This study
Δ*flaABCD*	MO6-24/O, Δ*flaABCD*, Km^R	This study
Δ*flaJ*	MO6-24/O, Δ*flaJ*, Km^R	This study
Δ*flhA*	MO6-24/O, Δ*flhA*	This study
flgK::Tn	MO6-24/O, *flgK::*mini-*Tn lacZ1*-Km^R	(2)
V. cholerae		
ATCC14033	Serovar O1, biotype ElTor, serotype Inaba	ATCC
V. parahaemolyticus		
RIMD2210633	Serotype O3:K6	ATCC/BAA-238
E. coli		
DH5α	f80d*lacZ* DM15 *recA1 endA1 gyrA96 relA1 thi-1 hsdR17*(r$_k^-$ m$_k^-$) *supE44 deoR Δ*(*lacZYA-argF*) *U169*	Laboratory collection
SM10λpir	*thi-1 thr leu tonA lacY supE recA*:: Rp4-2-Tc::Mu*λpir*, OriT of RP4, Km^R	(3)
JM109	*endA1 recA1 gyrA96 thi-1 hsdR17*(r$_k^-$ m$_k^-$) relA1 *supE44* (*lac-proAB*)[F'*traD3 6proAB lacI*^{q}*Z* M15]	Promega
BTH101	F$^-$ *cya-99 araD139 galE15 galK16 rpsL1* (*Str*^{r}) *hsdR2 mcrA1 mcrB1*	Euromedex
**Plasmids**		
pHK0011	pRK415 with promoterless *luxAB*, Tc^R	(4)
pHK0011-flaA	*flaA*::*luxAB* transcriptional fusion in pHK0011	This study
pHK0011-flaB	*flaB*::*luxAB* transcriptional fusion in pHK0011	This study
pHK0011-flaC	*flaC*::*luxAB* transcriptional fusion in pHK0011	This study
pHK0011-flaD	*flaD*::*luxAB* transcriptional fusion in pHK0011	This study
pHK0011-flaE	*flaE*::*luxAB* transcriptional fusion in pHK0011	This study

pHK0011-flaF	*flaF*::*luxAB* transcriptional fusion in pHK0011	This study
pBlueScript II SKII(+)	Cloning vector; Ap^{R} , *lac* promoter, f1, ColE1	Stratagene
pMflaE01	pBlueScript SKII(+) with 1,156-bp region of *flaE*	This study
pMflaE02	pMflaE01 with 1.2-kb kanamycin resistance gene, Km^{R}	This study
pMflaE03	pDM4 containing ApaI and SacI fragment of pMflaE02	This study
pMflaF01	pBlueScript SKII(+) with 780-bp upstream region of *flaF*	This study
pMflaF02	pMflaF01 with 910-bp downstream region of *flaF*	This study
pMflaF03	pMflaF02 with 1.2-kb kanamycin resistance gene, Km^{R}	This study
pMflaF04	pDM4 containing ApaI and SacI fragment of pMflaF03	This study
pMflaE_01	pBlueScript SKII(+) with 768-bp upstream region of *flaE*	This study
pMflaE_02	pMflaE_01 with 854-bp downstream region of *flaE*	This study
pMflaE_03	pDM4 containing SalI and SacI fragment of pMflaE_02	This study
pMflaB01	pBlueScript SKII(+) with 820-bp upstream region of *flaB*	This study
pMflaB02	pMflaB01 with 550-bp downstream region of *flaB*	This study
pMflaB03	pMflaB02 with 1.2-kb kanamycin resistance gene, Km^{R}	This study
pMflaB04	pDM4 containing ApaI and SacI fragment of pMflaB03	This study
pMflaD01	pBlueScript SKII(+) with 821-bp upstream region of *flaD*	This study
pMflaD02	pMflaD01 with 552-bp downstream region of *flaD*	This study
pMflaD03	pDM4 containing ApaI and XbaI fragment of pMflaD02	This study
pMflaC01	pBlueScript SKII(+) with 905-bp upstream region of *flaC*	This study
pMflaC02	pMflaC01 with 780-bp downstream region of *flaC*	This study
pMflaC03	pDM4 containing ApaI and SacI fragment of pMflaC02	This study
pMflaA01	pBlueScript SKII(+) with 780-bp upstream region of *flaA*	This study
pMflaA02	pMflaA01 with 600-bp downstream region of *flaA*	This study
pMflaA03	pDM4 containing SalI and XbaI fragment of pMflaA02	This study
pSKflaJ01	pBlueScript SKII(+) with 806-bp upstream region of *flaJ*	This study
pSKflaJ02	pSKflaJ01 with 651-bp downstream region of *flaJ*	This study
pKAS-flaJ	pKAS32 containing SalI and XbaI fragment of pSKflaJ02	This study
pYflhA01	pBlueScript SKII(+) with 590-bp upstream region of *flhA*	This study
pYflhA02	pYflhA01 with 890-bp downstream region of *flhA*	This study
pYflhA03	pYflhA02 with 1.2-kb kanamycin resistance gene, Km^{R}	This study
pYflhA04	pDM4 containing ApaI and SpeI fragment of pYflhA03	This study

pUC4K	pUC4 with *nptI*; Ap^R, Km^R	Pharmacia Biotech
pDM4	Suicide vector; *ori*R6K, Cm^R	(5)
pKAS32	Suicide vector; *rpsL*, Ap^R	(6)
pQE30	Expression vector, Ap^R	Qiagen
pQE30-FlaA	pQE30 containing 1,131-bp *V. vulnificus flaA* ORF	(7)
pQE30-FlaB	pQE30 containing 1,134-bp *V. vulnificus flaB* ORF	This study
pQE30-FlaC	pQE30 containing 1,158-bp *V. vulnificus flaC* ORF	This study
pQE30-FlaE	pQE30 containing 1,128-bp *V. vulnificus flaE* ORF	This study
pQE30-FlaF	pQE30 containing 1,134-bp *V. vulnificus flaF* ORF	This study
pQE30-FlaAvc	pQE30 containing 1,140-bp *V. cholerae flaA* ORF	This study
PQE30-FlaEvc	pQE30 containing 1,100-bp *V. cholerae flaE* ORF	This study
pQE30-FlaCvp	pQE30 containing 1,155-bp *V. parahaemolyticus flaC* ORF	This study
pQE30-FlaEvp	pQE30 containing 1,125-bp *V. parahaemolyticus flaE* ORF	This study
pQE30-FlaFvp	pQE30 containing 1,134-bp *V. parahaemolyticus flaF* ORF	This study
pRK415	IncP *ori*, broad-host-range vector *oriT* of RP4, Tc^R	(8)
pRK415-flaE	pRK415 with 1,135bp VVMO6_02251	This study
pRK415-flaF	pRK415 with 1,161bp VVMO6_00807	This study
pRK415-flhA	pRK415 with 2,103bp VVMO6_00832	This study
pRK415-12661	pRK415 with 1,403-bp VVMO6_01620 (in EPS-cluster I)	(9)
pRK415-21579	pRK415 with 2,313-bp VVMO6_03348 (in EPS-cluster II)	(9)
pRK415-12305	pRK415 with 2,285-bp VVMO6_01180 (in EPS-cluster III)	(9)
pKT25	BACTH plasmid for T25 fragment of adenylate cyclase (Km^r)	Euromedex
pUT18c	BACTH plasmid for T18 fragment of adenylate cyclase (Ap^r)	Euromedex
pKT25-*zip*	BACTH plasmid for T25 fragment fused to GCN4 leucine zipper	Euromedex
pUT18c-*zip*	BACTH plasmid for T18 fragment fused to GCN4 leucine zipper	Euromedex
pKT25-FlgL	pKT25, T25-*flgL* fusion	This study
pKT25-FliD	pKT25, T25-*fliD* fusion	This study
pKT25-FlaE	pKT25, T25-*flaE* fusion	This study

pKT25-FlaF	pKT25, T25-*flaF* fusion	This study
pKT25-FlaB	pKT25, T25-*flaB* fusion	This study
pUT18c-FlaB	pUT18c, T18-*flaB* fusion	This study
pUT18c-FlaE	pUT18c, T18-*flaE* fusion	This study
pUT18c-FlaF	pUT18c, T18-*flaF* fusion	This study

[1] **References**

1. Wright AC, Simpson LM, Oliver JD, Morris JG. 1990. Phenotypic evaluation of acapsular transposon mutants of *Vibrio vulnificus*. Infect Immun 58:1769-1773.

2. Kim HS, Lee MA, Chun SJ, Park SJ, Lee KH. 2007. Role of NtrC in biofilm formation via controlling expression of the gene encoding an ADP-glycero-manno-heptose-6-epimerase in the pathogenic bacterium, *Vibrio vulnificus*. Mol Microbiol 63:559-574.

3. Simon R, Priefer U, Puhler A. 1983. A broad host range mobilization system for *in vivo* genetic engineering: transposon mutagenesis in gram negative bacteria. Nat Biotechnol 1:784-791.

4. Jeong HS, Jeong KC, Choi HK, Park KJ, Lee KH, Rhee JH, Choi SH. 2001. Differential expression of *Vibrio vulnificus* elastase gene in a growth phase-dependent manner by two different types of promoters. J Biol Chem 27:13875-13880.

5. Milton DL, O'Toole R, HÖrstedt P, Wolf-Watz H. 1996. Flagellin A is essential for the virulence of *Vibrio anguillarum*. J Bacteriol 178:1310-1319.

6. Skorupski K, Taylor RK. 1996. Positive selection vectors for allelic exchange. Gene 169:47-52.

7. Lee HJ, Kim JA, Lee MA, Park SJ, Lee KH. 2013. Regulation of haemolysin (VvhA) production by ferric uptake regulator (Fur) in *Vibrio vulnificus*: repression of *vvhA* transcription by Fur and proteolysis of VvhA by Fur-repressive exoprotease. Mol Microbiol 88:813-826.

8. Keen NT, Tamaki S, Kobayashi D, Trollinger D. 1988. Improved broad-host-range plasmids for DNA cloning in Gram-negative bacteria. Gene 70:191-197.

9. Kim HS, Park SJ, Lee KH. 2009. Role of NtrC-regulated exopolysaccharides in the biofilm formation and pathogenic interaction of *Vibrio vulnificus*. Mol Microbiol 74:436-453.
