# Supplementary material for: Role of Flagellin-Homologous Proteins in Biofilm Formation by Pathogenic Vibrio Species
Source: mBio. 2019 Aug 13;10(4):e01793-19. doi: 10.1128/mBio.01793-19 (PMC6692518; doi:10.1128/mBio.01793-19)
Supplement: TABLE S3 [file mBio.01793-19-st003.pdf]

Table S3. Oligonucleotides used in this study

Strain/Gene [1]	Primer name	Sequence (5'->3'; Restriction sites underlined)
**For construction of deletion mutants**		
Δ*flaABCD*	flaA-upF	ACGCGTCGACGCTAGCGACGTACATCAACGGTC
	flaA-upR	AACTGCAGGCTGTGCGGTCATTGCTGACACG
	flaA-downF	AACTGCAGTCTTAGCTTGTTGGGCTAATACAG
	flaA-downR	GCTCTAGAGACAACTTTGCTGACCATGGAG
	flaB-upF	CCGCTCGAGTGCGAAAGCCGGCGATGATATCGA
	flaB-upR	CGGGATCCTCACTGCCATGATGAATTCTCC
	flaB-downF	CGGGATCCCTCAGCGCTAAGTCTACTAGGCT
	flaB-downR	CGAGCTCGATGTTGGTGGTCTCTGTCATTGC
	flaC-upF	CCCTCGAGCATTCACTGCCATGATGAATTCTCC
	flaC-upR	CGGGATCCCTGATTTTGCGAAGGAAACGACGC
	flaC-downF	CGGGATCCCGTTAGTGCTTACTGTTACAGCC
	flaC-downR	CGAGCTCTCGGTGTCTGATGCCAATGCC
	flaD-upF	ACGCGTCGACCTGTCTCCAGGCTTAAGCGAGATC
	flaD-upR	CGGGATCCGCCTACTAGGCTAATCTCTGAGC
	flaD-downF	CGGGATCCCGCTGTGCTGTCATTGCTGCTACG
	flaD-downR	GCTCTAGAGCTCTCTCATCTCTCACCTGC
Δ*flaEF*	flaE01-upF	ACGCGTCGACCGCAGAGCACTCTAAGTAATCTGC
	flaE01-upR	CGGGATCCGCCCCAGGCAGTGCAATTGGATTGC
	flaE01-downF	CGGGATCCGCCTCTGAGCGACCATCGCAGAC
	flaE01-downR	CGAGCTCGACATGCGCTCTGACAACGTG
	flaF-upF	CCGCTCGAGGCGCCTTCAACGGATGTGGATGTG
	flaF-upR	CGGGATCCCGGTGATCGCCACAACTCTTGGTCC
	flaF-downF	CGGGATCCGGGATAAGCGCCGGAGTGACTGG
	flaF-downR	CGAGCTCGCAATCGTCGTCAAGTCGTTGTCGCC
flaE::*nptI*	flaE-up	ACGCGTCGACGTGAGCAACCTAAGATAGCAATCC
	flaE-down	CGAGCTCGGAGAGGGATATCGATTAAATGG
Δ*flaF*	flaF-upF	CCGCTCGAGGCGCCTTCAACGGATGTGGATGTG
	flaF-upR	CGGGATCCCGGTGATCGCCACAACTCTTGGTCC

	flaF-downF	CGGGATCCGGGATAAGCGCCGGAGTGACTGG
	flaF-downR	CGAGCTCGCAATCGTCGTCAAGTCGTTGTCGCC
Δ*flaJ*	flaJ-upF	CGTCGACGATCCTACGACCGGTCAAAA
	flaJ-upR	CGGGATCCCCGCGCATAGTATTCCTCTT
	flaJ-downF	CGGGATCCGGCTGCGGATATTGGAATGT
	flaJ-downR	GCTCTAGAAAGAGGGTATTCTTACGTGC
Δ*flhA*	flhA-upF	AGTCGGGCCCGGCGGACGTTCCGACCGCGG
	flhA-upR	AACTGCAGGGCTGGTACTGAACGTTGGGGG
	flhA-downF	AACTGCAGGGCGACTCAGGAGCAAGAGCTC
	flhA-downR	GGACTAGTCGTCTTTCCACTTCCTGCCAC
**For construction of complementation plasmids**		
flaE	flaE-comF	CGGGATCCATGGTTTCACTCAATACCAACGTGTCTGC
	flaE-comR	GGGGTACCGAGCAACCTAAGATAGCAATCCAATTGC
flaF	flaF-comF	CCCAAGCTTCAGGACCAATATTTGTGGCG
	flaF-comR	CGGGATCCGTCACTCCGGCGCTTATCCC
flhA	flhA-comF	CCCAAGCTTATGGCTAAGAAATTTACTCTGCCG
	flhA-comR	CGGGATCCTCAGTTACCCACAGCCTGTAC
**For construction of *luxAB*-based transcription reporters**		
flaA	flaA_p-F	GGGGTACCCGTCGAAGAGCCGAATCAAAGATAC
	flaA_p-R	GCTCTAGAGCGGTCATTGCTGACACGTTAGTG
flaB	flaB_p-F	GGGGTACCCAGATTATGCGAAGGAAACCACGC
	flaB_p-R	GCTCTAGAGGTAACGCTGTGCTGTCATTGC
flaC	flaC_p-F	GGGGTACCCGCAGCTCGCATTTGGTAAAAC
	flaC_p-R	GCTCTAGAGCGGATACGTTAGTGCTTACTG
flaD/*E*	flaD_p-F	GGGGTACCGGCAGGTACTTCTATTCTTGCTC
	flaD_p-R	GCTCTAGACTGTGCTGTCATTGCTGCTACG
flaE	flaE_p-F	GGGGTACCGGACAACATCAACGAGAACGTGAAC
	flaE_p-R	GCTCTAGAGCGACCATCGCAGACACGTTGG
flaF	flaF_p-F	GGGGTACCGGGCTTAAGATTGTTCGTCACAGC
	flaF_p-R	GCTCTAGAGCGACAAGTGCTGCCACATTGG
**For construction of overexpression plasmids for recombinant proteins**		
rFlaA	flaAexp-F	CGGGATCCATGCGCGGTTCATTACAGGC

	flaAexp-R	CCCAAGCTTTCACGCCGCTGTATTAGC
rFlaB	flaBexp-F	CGGGATCCATGGCAGTGAATGTAAATACAAACG
	flaBexp-R	AACTGCAGTCTGTCTAGTTAAGGCGATTAGCC
rFlaC	flaCexp-F	CGGGATCCATGACTGCGCAACGTTATCTAAACAAAGCG
	flaCexp-R	AACTGCAGCAGACACAGGATTACTATTAGCCC
rFlaE	flaEexp-F	CGGGATCCATGGTTTCACTCAATACCAACG
	flaEexp-R	GGGGTACCGTGAGCAACCTAAGATAGCAATCC
rFlaF	flaFexp-F	CGGGATCCGTGGCGATCACCGTTAATACCAATGTGG
	flaFexp-R	AACTGCAGAGTCACTCCGGCGCTTATCCCAGC
rFlaA$_{vc}$	vcflaA-F	CGGGATCCATGACCATTAACGTAAATACC
	vcflaA-R	CCGAGCTCCTACTGCAATAACGAGATTGCAGAGTTTGG
rFlaE$_{vc}$	vcflaE-F	CGGGATCCATGGCCATGACGGTAAATACC
	vcflaE-R	CCCAAGCTTTTAATTACGCAGCAAAAACAGCAC
rFlaC$_{vp}$	vpflaC-F	CGGGATCCATGGCTGTAACAGTTAGTACTAACG
	vpflaC-R	CCCAAGCTTCTACAATAGTGACATTGC
rFlaE$_{vp}$	vpflaE-F	CGGGATCCATGGTCTCTTTAAATACCAATGTTGCCGC
	vpflaE-R	CCCAAGCTTTCAACTGAGCAAGCCTAGTGC
rFlaF$_{vp}$	vpflaF-F	CGGGATCCTTGGCTATCACCGTTAATACC
	vpflaF-R	CCCAAGCTTCTAGCCAAGCAAGGTAAGAGC
**For construction of Bacterial Two-Hybrid system plasmids**		
flaB	pKT25-FlaB-F	AACTGCAGGGATGGCAGTGAATGTAAATAC
	pKT25-FlaB-R	GATCGGTACCTTAGCCTAGTAGACTTAGCGCTG
flaE	pKT25-FlaE-F	GATCTCTAGAGATGGTTTCACTCAATACCATCGTG
	pKT25-FlaE-R	GATCGGTACCTAAGATAGCAATCCAATTGC
flaF	pKT25-FlaF-F	GATCTCTAGAGGTGGCGATCACCGTTAATACC
	pKT25-FlaF-R	GATCGGTACCTTATCCCAGCAAGGTCAACGC
flgL	pKT25-FlgL-F	GCTCTAGAGATGATTAGCCGTATCGCCAGTTTCCAC
	pKT25-FlgL-R	GGGGTACCCCGTCATCTCATTCAGAGACGGTTCG
fliD	pKT25-FliD-F	GCTCTAGAGATGAGTTTAGGCCCTTTGGGG
	pKT25-FliD-R	GGGGTACCGTCATGCGTTACTATCCCAGAGCG
flaB	pUT18c-FlaB-F	AACTGCAGTGGAGAATTCATCATGGCAGTG
	pUT18c-FlaB-R	CGGGATCCGTCTAGTTAAGGCGATTAGCC

flaE	pUT18c-FlaE-F	GATCTCTAGAGATGGTTTCACTCAATACCATCGTG
	pUT18c-FlaE-R	GATCGGTACCTAAGATAGCAATCCAATTGC
flaF	pUT18c-FlaF-F	GATCTCTAGAGGTGGCGATCACCGTTAATACC
	pUT18c-FlaF-R	GATCGGTACCTTATCCCAGCAAGGTCAACGC
**For RT-PCR of an *flaDE* transcript**		
flaD	flaD-F	CCATGCAATCAGCAACTTGGA
	flaD-R	TTAGCCTAGTAGGCTTAGCGCTG
flaE	flaE-F	ATGGTTTCACTCAATACCAACGTG
	flaE-R	TTTTTTTGGGTTTCAGCTAC

[1] Detailed procedures for mutant construction

(i) Construction of *flaABCD* deletion mutant. A *flaB* upstream region of 820-bp was amplified from the genomic DNA of *V. vulnificus* MO6-24/O using two primers, flaB-upF and flaB-upR. The PCR product was then cloned into a plasmid, pBlueScript SKII(+) to produce pMflaB01. A 550-bp DNA fragment containing downstream region of the *flaB* gene was made using primers flaB-downF and flaB-downR, and cloned into the corresponding sites of pMflaB01 to result in pMflaB02. Then, 1.2-kb kanamycin resistance gene was isolated from pUC4K (Pharmacia) and inserted into the BamHI site of pMflaB02 to produce pMflaB03. A 2,570-bp DNA fragment of pMflaB03 digested with ApaI and SacI was ligated to a suicide vector, pDM4 (1), to generate pMflaB04. *E. coli* SM10λ*pir* strain carrying pMflaB04 was conjugated with *V. vulnificus* MO6-24/O, and the exconjugants were selected on the thiosulfate citrate bile sucrose (TCBS) medium supplemented with 4 μg/ml chloramphenicol (2). Colonies with characteristics indicating a double homologous recombination event were isolated (resistance to 5% sucrose, sensitivity to chloramphenicol, and resistance to kanamycin) (2). Deletion of *flaB* gene in candidate colonies was confirmed by PCR with primers, flaB-upF and flaB-downR, and named Δ*flaB*.

A 821-bp PCR product containing the *flaD* upstream region was amplified using primers, flaD-upF and flaD-upR, and then cloned into pBlueScript SKII(+) to produce pMflaD01. A 552-bp PCR product was made to contain downstream region of *flaD* gene using flaD-downF and flaD-downR, and cloned into the corresponding sites of pMflaD01 to produce pMflaD02. The ApaI-XbaI DNA fragment of pMflaD02 was ligated into pDM4 to produce pMflaD03. The resultant plasmid in *E. coli* SM10λ*pir* strain was mobilized to Δ*flaB*, and the exconjugants were selected as described above. Colonies with characteristics indicating a double homologous recombination event were isolated as described above. Deletion of *flaD* gene in candidate colonies was confirmed by PCR with primers,

flaD-upF and flaD-downR, and named Δ*flaBD.*

A *flaC* upstream region of 905-bp was amplified using two primers, flaC-upF and flaC-upR. PCR product was cloned into pBlueScript SKII(+) to produce pMflaC01. A 780-bp DNA fragment containing downstream region of *flaC* gene was amplified using primers flaC-downF and flaC-downR, and cloned into the corresponding site of pMflaC01 to produce pMflaC02. A 1,190-bp DNA fragment of pMflaC02 digested with ApaI and SacI was ligated to pDM4, to generate pMflaC03. *E. coli* SM10λ*pir* strain carrying pMflaC03 was conjugated with Δ*flaBD* and the exconjugants were selected as described above. Colonies with characteristics indicating a double homologous recombination event were isolated as described above. Deletion of *flaC* gene in candidate colonies was confirmed by PCR with primers, flaC-upF and flaC-downR, and named Δ*flaBDC*.

A 780-bp PCR product containing the *flaA* upstream region was amplified using flaA-upF and flaA-upR, and then cloned into pBlueScript SKII(+) to produce pMflaA01. A 600-bp PCR product was made to contain downstream region of *flaA* gene using flaA-downF and flaA-downR, and cloned into the corresponding sites of pMflaA01 to produce pMflaA02. The SalI-XbaI DNA fragment of pMflaA02 was ligated into pDM4 to produce pMflaA03. The resultant plasmid in *E. coli* SM10λ*pir* strain was mobilized to Δ*flaBDC*, and the exoconjugants were selected as described above. Colonies with characteristics indicating a double homologous recombination event were isolated as described above. Deletion of *flaA* gene in candidate colonies was confirmed by PCR with primers, flaA-upF and flaA-downR, and named Δ*flaABDC*.

(ii) Construction of *flaE*::*nptI* mutant. A flanking region of the *flaE* (1,156-bp) was amplified from the genomic DNA of *V. vulnificus* MO6-24/O using the following two primers: flaE-up and flaE-down. The PCR product was the cloned in to pBlueScript SKII(+) to produce pMflaE01. A 1.2-kb kanamycin resistance gene was isolated from pUC4K and inserted into the PstI site of pMflaE01 to produce pMflaE02. About a 2.4-kb DNA insert from pMflaE02, digested with ApaI and SacI, was ligated to a suicide vector pDM4 to generate pMflaE03. An *E. coli* SM10λ*pir* strain carrying pMflaE03 was conjugated with *V. vulnificus* MO6-24/O and the exconjugants were selected on LBS medium supplemented with kanamycin. Colonies with characteristics indicating a double homologous recombination were further confirmed by PCR using flaE-up and flaE-down, and named strain *flaE*::*nptI*. For complementation of the *flaE* mutant, a 1,135-bp DNA fragment containing *V. vulnificus flaE* gene, which had been amplified using two primers, flaE-comF and flaE-comR, was cloned into a broad-host-range plasmid pRK415. The resultant plasmid, pRK415-flaE, was transformed into *E. coli* SM10λ*pir* and then transferred to *flaE*::*nptI V. vulnificus* by conjugation.

(iii) Construction of *flaF* deletion mutant. A 780-bp PCR product containing the *flaF* upstream region

was amplified using primers, flaF-upF and flaF-upR, and then cloned into pBlueScript SKII(+) to produce pMflaF01. A 910-bp PCR product was made to contain downstream region of *flaF* gene using flaF-downF and flaF-downR, and cloned into the corresponding sites of pMflaF01 to produce pMflaF02. *nptI* encoding a kanamycin resistance enzyme was isolated from pUC4K, and inserted into pMflaF02 to generate pMflaF03. The ApaI-SacI DNA fragment of pMflaF03 was ligated into pDM4 to produce pMflaF04. The resultant plasmid in *E. coli* SM10λ*pir* strain was mobilized to *V. vulnificus* MO6-24/O, and the exconjugants were selected as described above. Colonies with characteristics indicating a double homologous recombination event were isolated as described above. Deletion of *flaF* gene in candidate colonies was confirmed by PCR with primers, flaF-upF and flaF-downR, and named Δ*flaF*. For complementation of the *flaF* mutant, a 1,161-bp DNA fragment containing *V. vulnificus flaF* gene, which had been amplified using flaF-comF and flaF-comR, was cloned into pRK415. The resultant plasmid, pRK415-flaF, was transformed into *E. coli* SM10λ*pir* and then transferred to Δ*flaF V. vulnificus* by conjugation.

(iv) Construction of *flaEF* deletion mutant. A 768-bp PCR product containing the *flaE* upstream region was amplified using primers, flaE01-upF and flaE01-upR, and then cloned into pBlueScript SKII(+) to produce pMflaE_01. A 854-bp PCR product was made to contain downstream region of *flaE* gene using flaE01-downF and flaE01-downR, and cloned into the corresponding site of pflaE_01 to produce pMflaE_02. The SalI-SacI DNA fragment of pMflaE_02 was ligated into pDM4 to produce pMflaE_03. The resultant plasmid in *E. coli* SM10λ*pir* strain was mobilized to *V. vulnificus* Δ*flaF*, and exconjugants were then selected as described above. Colonies with characteristics indicating a double homologous recombination event were isolated as described above. Deletion of *flaE* gene in candidate colonies was confirmed by PCR with primers, flaE01-upF and flaE01-downR, and named Δ*flaEF*. For complementation of *flaE* or *flaF*, *E. coli* SM10λ*pir* containing pRK415-flaE or pRK415-flaF was mobilized to Δ*flaEF V. vulnificus* by conjugation, respectively.

(v) Construction of *flaJ* deletion mutant. A 806-bp *flaJ* upstream region (from -798 to +8 relative to its IC) was amplified from the genomic DNA of *V. vulnificus* MO6-24/O using primers, flaJ-upF and flaJ-upR. The PCR product was cloned into pBlueScript SKII(+) to produce pSKflaJ01. A DNA fragment containing 651-bp downstream of the *flaJ* ORF (from +390 to +1,040 relative to its IC) was generated using primers *flaJ*-downF and *flaJ*-downR, and cloned into the corresponding sites of pSKflaJ01 to produce pSKflaJ02. A 1,457-bp DNA fragment from pSKflaJ02, digested with SalI and XbaI, was ligated to a pKAS32 (3). pKAS-flaJ in *E. coli* SM10λ*pir* was mobilized to a *V. vulnificus*, and the conjugates were selected by plating the conjugation mixture of *E. coli* and *V. vulnificus* on LBS supplemented with 4 μg/ml of ampicillin. *V. vulnificus* colonies with characteristics of double homologous recombination event were further confirmed by PCR using primers, flaJ-upF and flaJ-

downR. The confirmed cell was named Δ*flaJ*.

(vi) Construction of *flhA* deletion mutant. A *flhA* upstream region of 590-bp was amplified from the genomic DNA of *V. vulnificus* MO6-24/O using two primers, flhA-upF and flhA-upR. The PCR product was then cloned into a plasmid, pBluescript SKII(+) to produce pYflhA01. A 890-bp DNA fragment containing downstream region of the *flhA* gene was made using primers flhA-downF and flhA-downR, and cloned into the corresponding sites of pYflhA01 to result in pYflhA02. Then, 1.2-kb kanamycin resistance gene was isolated from pUC4K, and inserted into the BamHI site of pYflhA02 to produce pYflhA03. A 2,680-bp DNA fragment of pYflhA03 digested with ApaI and SpeI was ligated to a suicide vector, pDM4, to generate pYflhA04. *E. coli* SM10λ*pir* strain carrying pYflhA04 was conjugated with *V. vulnificus* MO6-24/O, and the exconjugants were selected as described above. Candidate mutant colonies were confirmed by PCR using primers, flhA-upF and flhA-downR, and named Δ*flhA*. For complementation of the *flhA* mutant, a 2,103-bp DNA fragment containing *V. vulnificus flhA* gene, which had been amplified using two primers, flhA-comF and flhA-comR, was cloned into pRK415. The resultant plasmid, pRK415-flhA, was transformed into *E. coli* SM10λ*pir* and then transferred to Δ*flhA* by conjugation.

(vii) References

1. Milton DL, O'Toole R, HÖrstedt P, Wolf-Watz H. 1996. Flagellin A is essential for the virulence of *Vibrio anguillarum*. J Bacteriol 178:1310-1319.

2. Park KJ, Kang MJ, Kim SH, Lee HJ, Lim JK, Choi SH, Park SJ, Lee KH. 2004. Isolation and characterization of *rpoS* from a pathogenic bacterium, *Vibrio vulnificus* : role of σ^S in survival of exponential-phase cells under oxidative stress. J Bacteriol 186:3304-3312.

3. Skorupski K, Taylor RK. 1996. Positive selection vectors for allelic exchange. Gene 169:47-52.
